# Behavioral Profiling in Early Adolescence and Early Adulthood of Male Wistar Rats After Short and Prolonged Maternal Separation

**DOI:** 10.3389/fnbeh.2020.00037

**Published:** 2020-03-19

**Authors:** Stina Lundberg, Ingrid Nylander, Erika Roman

**Affiliations:** ^1^Research Group Neuropharmacology, Addiction and Behavior, Department of Pharmaceutical Biosciences, Uppsala University, Uppsala, Sweden; ^2^Division of Anatomy and Physiology, Department of Anatomy, Physiology and Biochemistry, Swedish University of Agricultural Sciences, Uppsala, Sweden

**Keywords:** adolescence, adult, behavior, early-life stress, handling, juvenile, maternal deprivation, multivariate concentric square field

## Abstract

Early-life stress and its possible correlations to genes, environment, and later health outcomes can only be studied retrospectively in humans. Animal models enable the exploration of such connections with prospective, well-controlled study designs. However, with the recent awareness of replicability issues in preclinical research, the reproducibility of results from animal models has been highlighted. The present study aims to reproduce the behavioral effects of maternal separation (MS) previously observed in the multivariate concentric square field^TM^ (MCSF) test. A second objective was to replicate the adolescent behavioral profiles previously described in the MCSF test. Male rats, subjected to short or prolonged MS or standard rearing, were subjected to behavioral testing in early adolescence and early adulthood. As seen in previous studies, the behavioral effects of MS in the MCSF were small at both tested time points. When tested in early adolescence, the animals exhibited a similar behavioral profile as previously seen, and the finding of adolescent behavioral types was also reproduced. The distribution of animals into the behavioral types was different than in the initial study, but in a manner consistent with developmental theories, as the current cohort was younger than the previous. Notably, the Shelter seeker behavioral type persisted through development, while the Explorer type did not. The lack of basal behavioral effect after MS is in line with the literature on this MS paradigm; the working hypothesis is that the prolonged MS gives rise to a phenotype predisposed to negative health outcomes but which is not apparent without additional provocation.

## Introduction

Early-life stress has evident adverse effects on several physiological systems, but the most emphasized is the increased vulnerability to develop psychiatric disorders (Ehlert, 2013; Maniam et al., 2014; Bick and Nelson, 2016). Various animal models have been developed to study this relationship, each with different types of stressors and developmental timings. Maternal separation (MS) is such a category of models that manipulates the early postnatal environment of the offspring. Prolonged MS, where the offspring is removed from its caretaker for between 3 and 6 h daily, is considered an early-life stress condition. However, the reported effects of prolonged MS vary considerably; some studies report increased impulsiveness/hyperactivity (Brake et al., 2004; Colorado et al., 2006), others increased anxiety- and/or depression-like behavior (Kalinichev et al., 2002; Jin et al., 2018), and yet others only limited or no effects (Shalev and Kafkafi, 2002; Marmendal et al., 2004; Farkas et al., 2009). Factors contributing to these discrepancies are probably differences in the MS methodology, model organism, the outcome assessment method and timing, and even the choice of control group (Lehmann and Feldon, 2000; Nylander and Roman, 2013; Vetulani, 2013).

Behavioral tests constitute an important part of animal models of psychiatric disorders (Brown and Bolivar, 2018; Bale et al., 2019). Recently, behavioral neuroscience has been mentioned as an example in the reproducibility crisis discussion (Kafkafi et al., 2018). Therefore, the choice of behavioral test and its validity and reproducibility have been emphasized. The multivariate concentric square field^TM^ (MCSF) test is an ethologically founded behavioral test that is unbiased by underlying mental condition, as it seeks to establish a behavioral profile of the tested animal (Meyerson et al., 2006). The MCSF has been validated for use in adult (Meyerson et al., 2006, 2013) and adolescent (Lundberg et al., 2019) rats. It has also been shown to have a greater sensitivity than classical behavioral tests in detecting effects of both pharmacological and non-pharmacological interventions (Roman et al., 2006; Meyerson et al., 2013; Karlsson and Roman, 2016).

The aim of the present study was to evaluate the behavioral outcome after prolonged MS compared to short MS and standard rearing at two developmental time points. Behavioral assessment was made with the MCSF test at 4 (early adolescence) and 9 (early adulthood) weeks of age, to study the short-term behavioral outcome and the effect of development.

## Materials and Methods

**FIGURE 1:**
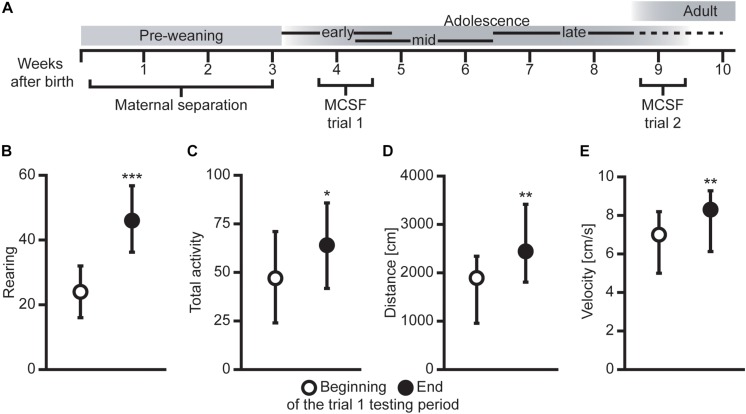
**(A)** Overview of the experimental outline with age in weeks and approximate developmental stages [adopted from Lundberg et al. (2019) with permission]. Difference in **(B)** rearing, **(C)** total activity (i.e., sum of all frequencies), **(D)** total distance traveled (cm), and (**E**) mean velocity (cm/s) between animals tested in the beginning (first half, *n* = 39) compared to the end (second half, *n* = 32) of the MCSF trial 1 testing period. Data presented as median with upper and lower quartiles. *P < 0.05, **P < 0.01, ***P < 0.001, Mann–Whitney *U* test.

An overview of the experimental timeline can be seen in Figure 1A, and a detailed description of the methods can be found in Comasco et al. (2015) regarding the MS procedure and in Meyerson et al. (2006) and Roman and Colombo (2009) for the MCSF procedure. Previously published results from the animal cohort (developmental body weights, voluntary ethanol consumption, and later genetic and epigenetic results) can be found in Bendre et al. (2015, 2018), Comasco et al. (2015); Nylander et al. (2016), Todkar et al. (2016); Granholm et al. (2017), and Vrettou et al. (2017).

In short, pregnant dams [*n* = 25, RccHan^TM^ WI, Harlan (currently Envigo), Horst, Netherlands] arrived at gestational day 15. Litters were sexed and cross-fostered on the day of birth [postnatal day (PND) 0] and randomized into one of three rearing conditions: daily separations on PND 1–21 for 360 (MS360) or 15 (MS15) minutes or kept according to standard animal facility rearing (AFR).

At PND 22, the litters were weaned, and only male offspring continued in the study (MS360, *n* = 30; MS15, *n* = 21; AFR, *n* = 21). The light cycle was reversed, and the animals were group housed three per cage with littermates.

In early adolescence (PND 26–31; 4–9 days after weaning), the animals were tested for the first time in the MCSF (trial 1). The second MCSF trial was performed 34–35 days later (at 61–65 days of age, i.e., in early adulthood). Both tests were performed during the dark period of the light/dark cycle.

### The MCSF Test

The MCSF arena is 100 cm × 100 cm with 10 defined zones: the center with central circle, three corridors used to move between the different parts of the arena, a covered shelter called the dark corner room (DCR), the slightly raised hurdle with hole board for nose poking, the slope leading up to the bridge entrance, and the illuminated bridge [for illustration see Lundberg et al. (2019)].

The animals were brought directly from their home cages and placed into the center of the arena, facing the wall not leading to a corridor. The trial started immediately as the animal was released into the arena and lasted for 20 min. The animals were recorded from above by a video camera and observed from an adjacent room. Direct observation was used to score number of rearing, grooming, and stretched attend postures (SAPs). After each trial, the number of urinations and fecal boli in the arena were counted, and the number of nose pokes in the hole board in the hurdle was read from a photocell counter. Score 3.3 (Soldis, Uppsala, Sweden) was used to manually score latency (*L*, s), frequency (*F*), and duration (*D*, s) of visits to the zones of the MCSF arena; EthoVision 2.3 (Noldus Inc., Wageningen, Netherlands) was used for automatic tracking of distance (total, cm) and velocity (mean, cm/s) in the arena. Additional parameters were derived as previously described (Lundberg et al., 2019).

### Statistical Analysis

Classical statistical analysis was carried out in Statistica 13 (TIBCO Software Inc., Tulsa, OK, United States). The data were examined for normality with Shapiro–Wilk’s test; the majority of the parameters were not normally distributed, and thus, non-parametric statistics were used. Between-group differences in continuous parameters were analyzed with Mann–Whitney *U* test (two initial groups) or Kruskal–Wallis ANOVA by ranks with *post hoc* Mann–Whitney *U* test (more than two initial groups); categorical parameters were analyzed with the maximum-likelihood chi-square test. Within-group differences in continuous parameters were analyzed with Wilcoxon-matched pairs test and categorical parameters with McNemar chi-square test. Effects sizes were calculated for significant differences according to Fritz et al. (2012).

Multivariate data analysis in the form of principal component analysis (PCA) was carried out in SIMCA 15 (Sartorius Stedim Data Analytics AB, Umeå, Sweden). Autofit was used to generate each model; this produces the maximum number of significant components. The components were inspected and excluded if they had eigenvalues <2 or large negative *Q*^2^ values. The latency, percental frequency, and percental duration parameters were not included in the PCA, and other parameters were excluded when advised by the software due to minimal variance.

#### Rank Order Procedures—Adult Trend Analysis and Adolescent Type Scores

Two rank-order procedures to summarize related parameters into compound variables were applied. For adult animals, the trend analysis (Meyerson et al., 2013) was used. It groups, ranks, and sums parameters into five different behavioral categories: general activity, exploratory activity, risk assessment, risk taking, and shelter seeking. However, the trend analysis has previously been shown to be invalid for animals tested in mid-adolescence, and instead, a separation of individuals into behavioral types called Explorers (characterized by high activity), Shelter seekers (high shelter-seeking behavior and low activity and risk-taking behavior), and Main type (intermediates) have been described (Lundberg et al., 2019). To quantify the outlying types, the same method as the trend analysis was applied on the significant parameters characterizing the behavioral types, creating compound variables called *type scores*. In the type score *exploration*, the following parameters are included: distance, velocity, and D/F center (negative relationship, ranking reversed before summarizing). In the type score *shelter seeking*, D DCR, D/F DCR, and nose pokes have a positive relationship with the behavioral type, and total activity, velocity, distance, D center, D bridge entrance, and D bridge have a negative relationship (Lundberg et al., 2019).

## Results

The behavioral profile of male MS360, MS15, and AFR offspring was assessed with the MCSF test at 4 (trial 1, early adolescence) and 9 (trial 2, early adulthood) weeks of age. One animal was excluded from trial 1 and three from trial 2 due to technical issues during testing. One animal was euthanized, due to malocclusion, between trials 1 and 2.

The effect of order in the two MCSF trials was examined by comparing the animals tested in the beginning (first half) of each testing period with the animals tested in the end (second half) of the corresponding testing period. As seen in Figures 1B–E, in trial 1, the activity was increased in animals tested in the end compared to the beginning of the testing period in all measures of activity, i.e., rearing, total activity, distance traveled, and velocity. Some additional activity-associated parameters were increased in the animals tested in the end compared to the beginning of the testing period in trial 1 (statistics not shown, Supplementary Data Sheet S1). This pattern was not seen in trial 2, where only a few mixed parameters were different between the animals tested in the beginning compared to the end of the testing period (statistics not shown, Supplementary Data Sheet S1).

### MCSF Performance by Rearing Condition

**FIGURE 2:**
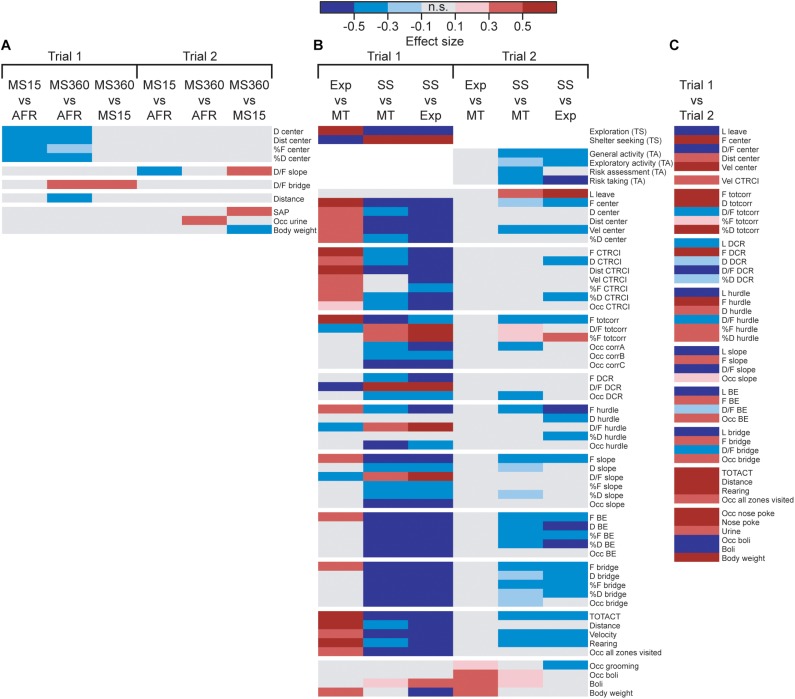
Heatmap of effect sizes for the significant differences comparing the **(A)** rearing conditions in trials 1 and 2 (MS15, *n*_trial__s__1,__2_ = 21; MS360, *n*_trial__1_ = 30, *n*_trial__2_ = 28; AFR, *n*_trial__1_ = 20, *n* = _trial 2_ = 19), **(B)** behavioral types in trials 1 and 2 (Explorers, *n*_trial__1_ = 9, *n*_trial__2_ = 8; Shelter seekers, *n*_trial__1,__2_ = 20; Main type *n*_trial__1_ = 42, *n*_trial__2_ = 39), and (**C**) trial 1 (*n* = 71) with trial 2 (*n* = 68) in the whole cohort. The parameters are sorted as followed: rank order procedures (type scores and trend analysis categories), zone measures (center, CTRCI, totcorr, DCR, hurdle, slope, BE, and bridge), activity parameters, and miscellaneous parameters. Parameters without any significant difference are not shown in the figure. AFR, animal facility reared; BE, bridge entrance; CTRCI, central circle; corr, corridor; D, duration; DCR, dark corner room; D/F, duration per visit; Dist, total distance; F frequency; MS15, maternal separation 15 min; MS360, maternal separation 360 min; n.s., non-significant; Occ, occurrence; SAP, stretched attend posture; TA, trend analysis; TOTACT, total activity; totcorr, total corridor; TS, type score; Vel, mean velocity.

The result from the MCSF tests analyzed by rearing condition can be seen in Figure 2A and Supplementary Tables S1, S2. Only a few parameters differed depending on the rearing condition; in trial 1, MS360 and MS15 rats behaved differently in the center zone of the MCSF (lower duration, duration per visit, and percental duration and frequency) compared to AFR animals. MS360 rats had higher duration per visit on the bridge than both MS15 and AFR rats and had lower distance traveled than AFR rats. No difference in the type scores was observed among the rearing conditions.

In trial 2 (Figure 2A and Supplementary Table S2), MS15 rats had lower duration per visit to the slope than MS360 and AFR rats, MS360 rats performed more SAPs than MS15 rats, and a higher proportion of MS360 rats urinated in the arena compared to AFR rats. At the second trial, MS360 rats weighed less than the MS15 rats; no other body weight difference among the different rearing conditions was detected. No difference in the trend analysis categories was observed among the rearing conditions.

**FIGURE 3:**
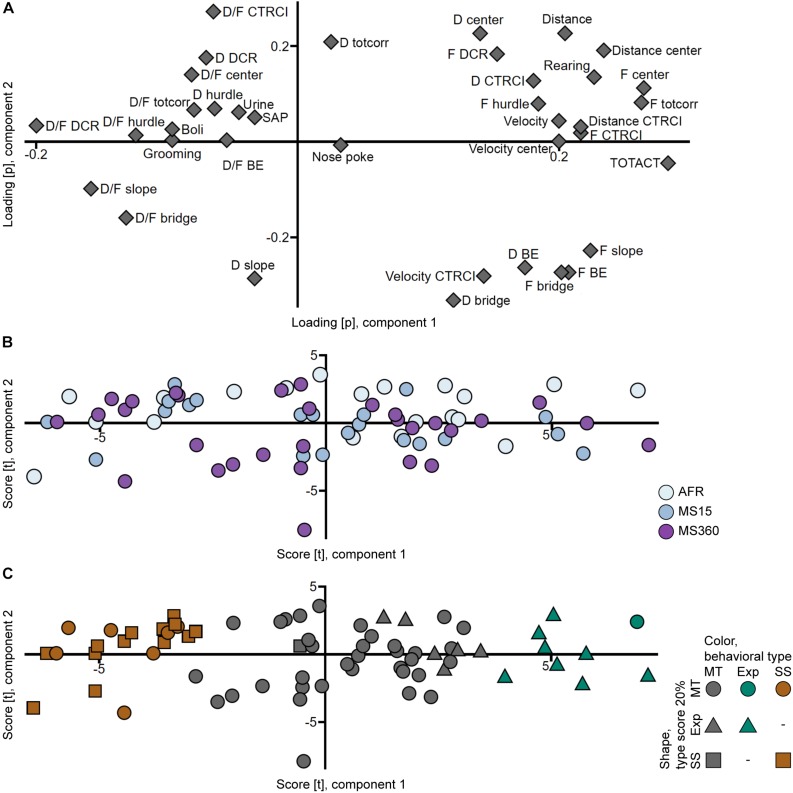
Scatter plots of the **(A)** parameter loadings and **(B,C)** individual scores from the adolescent PCA (*n* = 71, two components, *R*^2^*X* = 0.439, *Q*^2^ = 0.314). **(B)** is colored according to rearing condition and **(C)** according to behavioral type, and the shapes show the top 20% of each type score (exploration and shelter seeking). AFR, animal facility reared; BE, bridge entrance; CTRCI, central circle; D, duration; DCR, dark corner room; D/F, duration per visit; Exp, Explorer/exploration; F, frequency; MS15, maternal separation 15 min; MS360, maternal separation 360 min; MT, Main type; SAP, stretched attend posture; SS, Shelter seeker/seeking; TOTACT, total activity; totcorr, total corridor.

**FIGURE 4:**
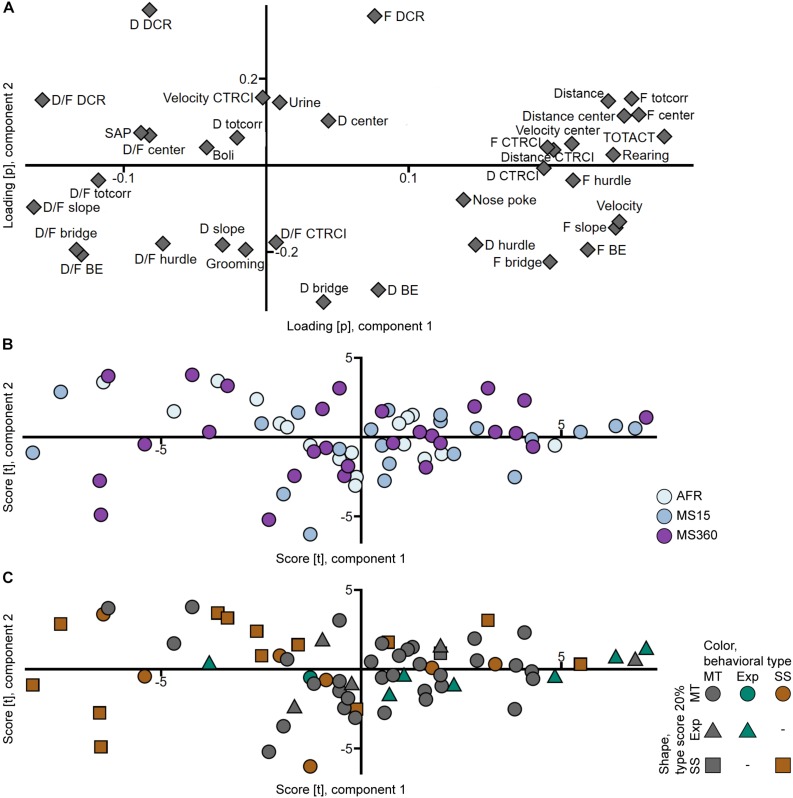
Scatter plots of the **(A)** parameter loadings and **(B,C)** individual scores from the adult PCA (*n* = 67, two components, *R*^2^*X* = 0.447, *Q*^2^ = 0.290). **(B)** is colored according to rearing condition and **(C)** according to behavioral type, and the shapes show the top 20% of each type score (exploration and shelter seeking). AFR, animal facility reared; BE, bridge entrance; CTRCI, central circle; D, duration; DCR, dark corner room; D/F, duration per visit; Exp, Explorer/exploration; F, frequency; MS15, maternal separation 15 min; MS360, maternal separation 360 min; MT, Main type; SAP, stretched attend posture; SS, Shelter seeker/seeking; TOTACT, total activity; totcorr, total corridor.

That the rats from the different rearing conditions have similar behavioral profiles can also be seen in the PCA analyses (Figures 3A,B, 4A,B) where the individuals in the three rearing conditions are evenly spread out in the score plots both when tested in early adolescence (Figure 3B) and early adulthood (Figure 4B).

### MCSF Performance by Behavioral Type

The top 20% individuals in each type score were identified in the trial 1 PCA score plot (Figure 3C), with the aim to replicate the finding of the behavioral types from Lundberg et al. (2019). Corresponding clusters were found but with different proportions; the 20% of individuals with the highest shelter seeking score loaded together but with additional individuals interspersed; only one individual with high shelter seeking score loaded away from this cluster. On the other hand, the 20% of individuals with the highest exploration score did not load together; some loaded separate from the Main type cluster, but several loaded intermingled with the main body of individuals. These clusters were used to classify the animals into the behavioral types Shelter seekers (20 individuals, 28%), Explorers (9 individuals, 13%), and Main type (42 individuals, 59%).

The behavioral types differed in their type scores, with Explorers having higher exploration score than Main type and Shelter seekers having lower exploration score than both Explorers and Main types. The reverse was seen for the shelter seeking score; Shelter seekers had higher shelter seeking score than both Explorers and Main type, while Explorers scored lower than the Main type (Figure 2B and Supplementary Table S3). The rearing condition did not affect which behavioral type an individual was classified as (Supplementary Table S1). However, a higher proportion of the individuals tested in the beginning of the trial 1 testing period was classified as Shelter seekers and fewer as Explorers than among the individuals tested at the end of the period (Supplementary Table S3).

When analyzing trial 1 by behavioral type, profound differences were found (see Figure 2B and Supplementary Table S3); 46 of the 75 parameters had some behavioral-type-dependent difference. Explorers had higher activity in the arena in general (higher total activity, distance, velocity, rearing, and a larger proportion that visited all zones of the arena) and in the central parts (higher frequency, duration, distance, and velocity at the center and central circle, and higher proportion that visited the central circle) than Main type animals. Explorers had higher number of visits than the Main type animals to all zones except the DCR and had lower average duration in the corridors, DCR, hurdle, and slope. Shelter seekers, on the other hand, had lower activity in the arena (lower total activity, distance, velocity, rearing, and a smaller proportion that visited all zones of the arena) than both Main type and Explorers and lower activity in the central parts of the arena (lower frequency, duration, and distance at the center and central circle) and on the entire bridge construction (lower frequency, duration, and proportion visiting the slope, bridge entrance, and bridge) than the other types. Instead, Shelter seekers had longer average visits to the corridors, DCR, hurdle, and slope, as well as fewer visits to the corridors in absolute numbers; but when correcting for the lower number of zone transits, they did percentage-wise more visits to the corridors than Main types and Explorers.

In trial 2, the Explorer behavioral type did not persist, while the Shelter seeker profile can be identified in the classical statistical analysis (Figure 2B and Supplementary Table S4), although with lower effect sizes and the clustering in the PCA score plot (Figure 4C) is not visible. However, in the PCA, most of the Shelter seekers still tend to load with lower contribution of component 1 than the other individuals (Figure 4C). In the trend analysis (Figure 2B and Supplementary Table S4), Explorers did not differ from the Main type animals, while Shelter seekers had lower general activity, exploratory activity, and risk taking than both Main type and Explorers, and lower risk assessment than Main type animals. Notably, the trend analysis category shelter seeking did not differ between the behavioral types.

### MCSF Performance With Repeated Testing

The effect of repeated testing/development can be seen in Figure 2C and Supplementary Table S5. The analysis was conducted with rearing conditions collapsed since the effect of rearing condition was small in both trials. Over time, the activity increased (higher total activity, distance, rearing, and proportion visiting all zones) and the latency to leave the center and latency to the other zones (except the central circle) were lower in trial 2 than in trial 1. The duration in the corridors and in the hurdle increased, the time spent in the DCR decreased, and the duration in the other zones were unchanged between the trials. The average duration decreased between trials 1 and 2 in all zones, except the central circle where it was unchanged. In the second trial, more individuals performed nose pokes, and the number of nose pokes also increased. More urinations but fewer defecations were observed, and the proportion of individuals defecating also decreased in trial 2 relative to trial 1.

## Discussion

The present study describes the MCSF behavioral profiles at 4 and 9 weeks of age in male rats subjected to different rearing conditions in the preweaning period. Only minor differences were found depending on the rearing condition, which replicates previous studies in both adolescent (Palm et al., 2013) and adult (Roman et al., 2006) male rats tested in the MCSF after MS. However, within the large individual variation in the test in early adolescence, it was possible to identify the same behavioral types as have been previously reported for adolescent animals (Lundberg et al., 2019). The present study advances the behavioral type concept further by developing, in correspondence to the trend analysis for adult animals (Meyerson et al., 2013), compound variables quantifying these behavioral types with the so-called type scores. This procedure will hopefully aid the interpretation of future studies, especially studies with a smaller number of animals, where the identification of the behavioral types can be more difficult as the number of animals in each behavioral type decreases.

The detected rearing-condition-dependent differences were few and are therefore difficult to interpret and draw conclusions from. However, the significant parameters are associated with some of the same behavioral categories as the previously described differences: risk taking in adolescent animals (Palm et al., 2013) and risk assessment in adults (Roman et al., 2006). What can be concluded from the present study is that, in early adolescence, MS males, independent of separation length, seem to have an avoidance of open areas, as indicated by the lower duration, distance, and percental duration and frequency to the center of the MCSF arena. This is in agreement with, for the MS360 animals, previous findings of behavior in the elevated plus maze (Ploj et al., 2002). However, as this is not supported by a corresponding difference among central circle parameters in the present study, the interpretation becomes less clear, and thus, the difference could also indicate decreased thigmotaxic behavior in both short and prolonged MS animals relative to AFR.

The behavioral effects of MS vary considerably between studies in both adolescence and adulthood; prolonged MS has been associated with as disparate behavioral characteristics as impulsiveness/hyperactivity (Brake et al., 2004; Colorado et al., 2006) and high anxiety- and/or depression-like behavior (Kalinichev et al., 2002; Jin et al., 2018), but no or small behavioral effects have also been reported (Shalev and Kafkafi, 2002; Marmendal et al., 2004; Farkas et al., 2009). These discrepancies can be due to the different behavioral tests used (Hånell and Marklund, 2014) and methodological differences in the MS protocols (Lehmann and Feldon, 2000; Pryce and Feldon, 2003; Nylander and Roman, 2012, 2013; Vetulani, 2013). Banqueri et al. (2017) suggested, in a direct comparison between different developmental lengths of separation, that MS limited to the first half of the preweaning period is responsible for the impulsive behavioral outcome, while MS throughout the preweaning period leads to an anxiety-like behavioral profile. Although, in our hands, using a consistent MS paradigm with separations throughout the preweaning period, we have consistently seen minor basal behavioral effects in male offspring, independent of testing age and behavioral test (Ploj et al., 2002; Roman et al., 2006; Palm et al., 2013; Lundberg et al., 2017). This is in line with the working hypothesis that this type of MS (daily 360 min separations throughout the preweaning period) leads to a phenotype predisposed to develop stress-related disorders but not overt changes in physiology and behavior without additional provocations (Nylander and Roman, 2012; Palm et al., 2013). This is supported by findings of dysregulated hypothalamic pituitary adrenal (HPA) axis reactivity in both young (Lundberg et al., 2017) and adult (Roman et al., 2006) MS360 males. However, the dysregulation seems to differ between young and adult individuals; shortly after weaning, MS360 males have increased baseline and recovery corticosterone levels (Lundberg et al., 2017), while in adulthood, it is stress-induced corticosterone levels that are blunted (Roman et al., 2006).

The fact that the identification of behavioral types in adolescent animals in the MCSF could be replicated from Lundberg et al. (2019) lends further support for this finding. In the present study, the proportion of the behavioral types differs from the initial finding; this is, however, not surprising since the present cohort is 1–2 weeks younger than in the previous study, and a higher proportion of Shelter seekers in a younger cohort is in agreement with behavioral developmental theories (Yurgelun-Todd, 2007; Doremus-Fitzwater et al., 2010; Sturman and Moghaddam, 2011). The Shelter seekers identified in early adolescence were still separable from the rest of the population in early adulthood, while the Explorer behavioral type was not. These results, together with the finding that Shelter seekers in early adulthood differed in all the trend analysis categories except precisely shelter seeking, are interesting new findings regarding how individual behavioral profiles develop with maturation. Important to note is that the trend analysis category shelter seeking only include DCR-related parameters, while the behavioral type encompass both increased shelter-seeking behavior as well as decreased activity and risk-taking behavior (Lundberg et al., 2019). That only the activity- and risk-associated differences characterizing Shelter seekers persists throughout adolescence is noteworthy and an interesting phenomenon for continued study.

What should be noted in the present study is that the light/dark cycle reversal together with weaning preceded the behavioral test in early adolescence with only 4–9 days. It is likely that these procedures affected the animals to some degree and may have played a part in the detected differences between the animals tested in the beginning and at the end of the trial 1 testing period, together with the age of the animal.

In the present study, it is impossible to detangle what differences between trials 1 and 2 that are age-dependent and what are due to repeated testing and thus established memory of the arena. Still, there were more differences in the present study between trials 1 and 2 than between individuals tested repeatedly with 1 week intertest interval in adolescence (Lundberg et al., 2019). This indicates that the present finding could be more due to developmental effects since the memory effect can be assumed to be stronger with 1 week intertest interval than with 5 weeks, even though the memory effect in adulthood has been described with testing intervals between 1 and 6 weeks (Meyerson et al., 2006; Roman et al., 2007; Karlsson et al., 2009; Roman and Colombo, 2009; Magara et al., 2015).

The effect of age in the MCSF has only been examined in a few previous studies. A comparison between males aged 5–6 weeks (mid-adolescence) and a literature cohort of males aged 10–11 weeks (early adulthood) (Lundberg et al., 2019) found clear differences between the age groups. However, the specific findings are partially contradicting to the present; mainly, the increase in activity seen in the present study is not evident in the previous work (Lundberg et al., 2019). This could, however, be attributed to the memory effect of repeated testing as discussed above. Another study, more similar to the present, found only minor-age-dependent differences following repeated testing in the MCSF after MS at week 4 (early adolescence) and week 15 (adulthood) of age (Palm et al., 2013). However, in Palm et al. (2013), the animals were housed individually during the intertest interval, which has been demonstrated to have a profound influence on behavior (Haller et al., 2014) and adolescent social isolation has also been shown to interact with MS (Biggio et al., 2014), possibly altering the behavioral outcome. It is also possible that the discrepancies between these three studies are due, in part, to an inverted u-shaped developmental trajectory making early adolescents and fully adult individuals more similar in their behavior than the intermediate developmental stages.

In conclusion, the present study replicates previous findings both regarding behavior in the MCSF after MS and adolescent behavior in the MCSF in general. The study also adds new information about developmental effects on behavior as reflected by the performance in the MCSF test.

## Data Availability Statement

All datasets generated for this study are included in the article/Supplementary Material.

## Ethics Statement

The animal study was reviewed and approved by the Uppsala Animal Ethical Committee in accordance with the Swedish Legislation on Animal Experimentation (Animal Welfare Act SFS1998:56) and the European Communities Council Directive (86/609/EEC).

## Author Contributions

ER and IN contributed to conceptualization and design of the study and provided the funding. SL performed the statistical analysis and wrote the first draft of the manuscript. All authors contributed to manuscript revision and read and approved the submitted version.

## Conflict of Interest

The authors declare that the research was conducted in the absence of any commercial or financial relationships that could be construed as a potential conflict of interest.
