# Supplementary material for: Behavioral Profiling in Early Adolescence and Early Adulthood of Male Wistar Rats After Short and Prolonged Maternal Separation
Source: Front Behav Neurosci. 2020 Mar 19;14:37. doi: 10.3389/fnbeh.2020.00037 (PMC7096550; doi:10.3389/fnbeh.2020.00037)
Supplement: Supplementary file 2 [file Table_1.DOCX]

Supplementary Table 1. Results from the first MCSF trial in animals reared according to the AFR (n=20), MS15 (n=21) or MS360 (n=30) rearing condition.

|  |  | **AFR** | | | | **MS15** | | | | | **MS360** | | | | |
| --- | --- | --- | --- | --- | --- | --- | --- | --- | --- | --- | --- | --- | --- | --- | --- |
|  |  | Median | Quartiles | | | Median | Quartiles | | | p-value | Median | Quartiles | | | p-value |
| **Type score** | Exploration | 115.5 | 82.0 | - | 146.3 | 116.0 | 82.0 | - | 149.0 |  | 98.5 | 64.0 | - | 140.0 |  |
|  | Shelter seeking | 328.5 | 232.8 | - | 420.3 | 325.5 | 278.5 | - | 451.0 |  | 351.3 | 298.0 | - | 403.0 |  |
| **Behavioral type** | Explorers | 3/20 |  |  |  |  | 3/21 |  |  |  | 3/30 |  |  |  |  |
|  | Shelter seekers | 5/20 |  |  |  |  | 7/21 |  |  |  | 8/30 |  |  |  |  |
|  | Main types | 12/20 |  |  |  |  | 11/21 |  |  |  | 19/30 |  |  |  |  |
| **Center** | L leave | 31.4 | 12.3 | - | 60.8 | 17.6 | 7.4 | - | 37.9 |  | 24.4 | 10.8 | - | 41.2 |  |
|  | F center | 10.5 | 5.5 | - | 13.0 | 7.0 | 5.0 | - | 11.0 |  | 6.5 | 3.0 | - | 12.0 |  |
|  | D center | 148.1 | 94.2 | - | 208.6 | 87.9 | 63.7 | - | 112.7 | * | 90.2 | 49.2 | - | 141.5 | * |
|  | D/F center | 14.1 | 10.8 | - | 19.5 | 10.6 | 9.7 | - | 12.5 |  | 11.1 | 8.5 | - | 17.7 |  |
|  | Distance center | 1631.8 | 1007.6 | - | 2215.3 | 967.7 | 710.4 | - | 1276.9 | * | 1016.4 | 501.6 | - | 1464.0 | * |
|  | Velocity center | 9.2 | 8.4 | - | 10.4 | 9.6 | 6.9 | - | 11.2 |  | 8.6 | 7.1 | - | 10.8 |  |
|  | %F center | 19.0 | 14.2 | - | 21.4 | 14.1 | 12.3 | - | 16.1 | * | 14.0 | 10.8 | - | 18.3 | * |
|  | %D center | 12.3 | 7.8 | - | 17.3 | 7.3 | 5.3 | - | 9.4 | * | 7.5 | 4.1 | - | 11.8 | * |
| **Central circle** | L CTRCI | 201.3 | 112.0 | - | 305.6 | 433.5 | 108.0 | - | 608.6 |  | 184.8 | 45.1 | - | 471.7 |  |
|  | F CTRCI | 2.0 | 0.5 | - | 2.5 | 1.0 | 0.0 | - | 2.0 |  | 1.0 | 0.0 | - | 2.0 |  |
|  | D CTRCI | 3.1 | 0.5 | - | 6.2 | 2.0 | 0.0 | - | 3.4 |  | 2.1 | 0.0 | - | 3.5 |  |
|  | D/F CTRCI | 1.6 | 1.4 | - | 2.6 | 1.7 | 1.4 | - | 2.1 |  | 1.4 | 1.1 | - | 2.5 |  |
|  | Distance CTRCI | 30.7 | 2.4 | - | 51.2 | 5.5 | 0.0 | - | 50.5 |  | 21.3 | 0.0 | - | 39.3 |  |
|  | Velocity CTRCI | 10.2 | 8.4 | - | 14.2 | 13.3 | 7.4 | - | 18.0 |  | 12.5 | 10.4 | - | 18.4 |  |
|  | %F CTRCI | 2.6 | 1.0 | - | 4.3 | 1.5 | 0.0 | - | 2.8 |  | 2.4 | 0.0 | - | 5.4 |  |
|  | %D CTRCI | 0.3 | 0.0 | - | 0.5 | 0.2 | 0.0 | - | 0.3 |  | 0.2 | 0.0 | - | 0.3 |  |
|  | Occ CTRCI | 15/20 |  |  |  | 13/21 |  |  |  |  | 20/30 |  |  |  |  |
| **Total corridor** | F total corr | 20.5 | 14.0 | - | 26.5 | 19.0 | 14.0 | - | 25.0 |  | 16.0 | 9.0 | - | 27.0 |  |
|  | D total corr | 360.1 | 303.3 | - | 407.1 | 276.2 | 236.2 | - | 385.5 |  | 293.7 | 173.2 | - | 354.5 |  |
|  | D/F total corr | 17.8 | 10.7 | - | 21.7 | 14.8 | 11.8 | - | 20.9 |  | 14.3 | 11.3 | - | 19.7 |  |
|  | %F total corr | 38.6 | 33.7 | - | 45.0 | 36.8 | 29.6 | - | 42.9 |  | 33.5 | 29.8 | - | 38.5 |  |
|  | %D total corr | 30.0 | 25.2 | - | 33.8 | 23.0 | 19.6 | - | 32.1 |  | 24.4 | 14.4 | - | 29.5 |  |
|  | Occ corrA | 17/20 |  |  |  | 18/21 |  |  |  |  | 23/30 |  |  |  |  |
|  | Occ corrB | 17/20 |  |  |  | 18/21 |  |  |  |  | 24/30 |  |  |  |  |
|  | Occ corrC | 17/20 |  |  |  | 20/21 |  |  |  |  | 25/30 |  |  |  |  |
| **Dark corner**  **room** | L DCR | 199.0 | 75.1 | - | 635.9 | 202.7 | 109.0 | - | 664.5 |  | 159.7 | 28.8 | - | 353.3 |  |
|  | F DCR | 4.0 | 2.5 | - | 6.5 | 5.0 | 2.0 | - | 6.0 |  | 4.5 | 1.0 | - | 7.0 |  |
|  | D DCR | 185.1 | 95.7 | - | 364.9 | 186.5 | 56.4 | - | 488.0 |  | 168.6 | 27.6 | - | 376.3 |  |
|  | D/F DCR | 31.8 | 26.8 | - | 106.7 | 62.2 | 25.8 | - | 72.0 |  | 41.8 | 28.8 | - | 67.1 |  |
|  | %F DCR | 6.2 | 4.5 | - | 10.6 | 6.1 | 3.5 | - | 12.0 |  | 6.9 | 2.9 | - | 10.7 |  |
|  | %D DCR | 15.4 | 7.9 | - | 30.4 | 15.5 | 4.7 | - | 40.6 |  | 14.0 | 2.3 | - | 31.3 |  |
|  | Occ DCR | 16/20 |  |  |  | 17/21 |  |  |  |  | 23/30 |  |  |  |  |
| **Hurdle** | L hurdle | 281.6 | 60.6 | - | 384.8 | 120.9 | 45.8 | - | 270.2 |  | 219.2 | 98.3 | - | 331.0 |  |
|  | F hurdle | 5.0 | 3.0 | - | 6.0 | 5.0 | 4.0 | - | 7.0 |  | 3.0 | 2.0 | - | 6.0 |  |
|  | D hurdle | 96.7 | 59.4 | - | 149.4 | 79.9 | 61.6 | - | 174.8 |  | 80.3 | 61.7 | - | 101.2 |  |
|  | D/F hurdle | 20.6 | 14.9 | - | 31.8 | 17.2 | 13.8 | - | 46.8 |  | 20.1 | 14.1 | - | 30.9 |  |
|  | %F hurdle | 8.0 | 4.5 | - | 10.3 | 7.0 | 5.6 | - | 11.8 |  | 7.6 | 5.3 | - | 10.4 |  |
|  | %D hurdle | 8.0 | 4.9 | - | 12.4 | 6.6 | 5.1 | - | 14.5 |  | 6.7 | 5.1 | - | 8.4 |  |
|  | Occ hurdle | 16/20 |  |  |  | 18/21 |  |  |  |  | 29/30 |  |  |  |  |
| **Slope** | L slope | 233.4 | 132.4 | - | 426.9 | 130.0 | 62.2 | - | 389.7 |  | 118.8 | 51.7 | - | 268.0 |  |
|  | F slope | 7.0 | 2.5 | - | 11.5 | 8.0 | 3.0 | - | 11.0 |  | 9.0 | 4.0 | - | 12.0 |  |
|  | D slope | 99.2 | 46.0 | - | 151.5 | 129.2 | 54.0 | - | 159.9 |  | 144.5 | 79.2 | - | 263.6 |  |
|  | D/F slope | 11.7 | 9.8 | - | 17.0 | 13.3 | 10.8 | - | 18.0 |  | 16.0 | 12.5 | - | 24.7 |  |
|  | %F slope | 11.8 | 7.2 | - | 15.8 | 12.9 | 9.1 | - | 18.4 |  | 15.1 | 8.4 | - | 19.0 |  |
|  | %D slope | 8.2 | 3.8 | - | 12.6 | 10.7 | 4.5 | - | 13.3 |  | 12.0 | 6.6 | - | 21.9 |  |
|  | Occ slope | 16/20 |  |  |  | 17/21 |  |  |  |  | 24/30 |  |  |  |  |
| **Bridge**  **entrance** | L BE | 316.0 | 232.8 | - | 450.3 | 271.9 | 122.8 | - | 459.8 |  | 264.6 | 106.1 | - | 340.5 |  |
|  | F BE | 4.0 | 0.0 | - | 9.5 | 7.0 | 0.0 | - | 12.0 |  | 7.0 | 0.0 | - | 11.0 |  |
|  | D BE | 25.5 | 0.0 | - | 48.1 | 43.8 | 0.0 | - | 67.4 |  | 35.4 | 0.0 | - | 65.3 |  |
|  | D/F BE | 5.8 | 3.5 | - | 9.6 | 6.1 | 4.0 | - | 7.7 |  | 5.7 | 4.3 | - | 8.1 |  |
|  | %F BE | 6.1 | 0.0 | - | 12.2 | 11.3 | 0.0 | - | 16.5 |  | 10.7 | 0.0 | - | 15.8 |  |
|  | %D BE | 2.1 | 0.0 | - | 4.0 | 3.6 | 0.0 | - | 5.6 |  | 2.9 | 0.0 | - | 5.4 |  |
|  | Occ BE | 14/20 |  |  |  | 15/21 |  |  |  |  | 22/30 |  |  |  |  |
| **Bridge** | L bridge | 350.2 | 258.5 | - | 624.5 | 302.9 | 145.0 | - | 508.3 |  | 297.7 | 133.6 | - | 345.1 |  |
|  | F bridge | 1.5 | 0.0 | - | 4.0 | 3.0 | 0.0 | - | 6.0 |  | 3.0 | 0.0 | - | 5.0 |  |
|  | D bridge | 50.8 | 0.0 | - | 159.5 | 91.4 | 0.0 | - | 214.6 |  | 148.3 | 0.0 | - | 268.0 |  |
|  | D/F bridge | 33.2 | 27.6 | - | 48.6 | 34.3 | 28.5 | - | 40.2 |  | 53.4 | 33.3 | - | 67.0 | *. + |
|  | %F bridge | 2.6 | 0.0 | - | 5.4 | 4.0 | 0.0 | - | 8.2 |  | 4.2 | 0.0 | - | 7.4 |  |
|  | %D bridge | 4.2 | 0.0 | - | 13.2 | 7.6 | 0.0 | - | 17.8 |  | 12.3 | 0.0 | - | 22.3 |  |
|  | Occ bridge | 13/20 |  |  |  | 14/21 |  |  |  |  | 21/30 |  |  |  |  |
| **Activity** | TOTACT | 57.0 | 30.0 | - | 78.0 | 57.0 | 32.0 | - | 75.0 |  | 56.0 | 32.0 | - | 80.0 |  |
|  | Distance total | 3006.2 | 1932.5 | - | 3736.1 | 2144.8 | 1619.6 | - | 2506.2 |  | 1779.9 | 1162.4 | - | 2236.4 | * |
|  | Velocity mean | 7.4 | 6.2 | - | 8.7 | 7.3 | 5.9 | - | 9.2 |  | 6.5 | 5.4 | - | 8.6 |  |
|  | Rearing | 35.5 | 18.5 | - | 56.0 | 30.0 | 23.0 | - | 50.0 |  | 29.0 | 20.0 | - | 46.0 |  |
|  | Occ all zones visited | 12/20 |  |  |  | 11/21 |  |  |  |  | 12/30 |  |  |  |  |
| **Miscellaneous** | Occ nose poke | 6/20 |  |  |  | 7/21 |  |  |  |  | 5/30 |  |  |  |  |
|  | Nose poke | 0.0 | 0.0 | - | 1.0 | 0.0 | 0.0 | - | 4.0 |  | 0.0 | 0.0 | - | 0.0 |  |
|  | Occ grooming | 12/20 |  |  |  | 16/21 |  |  |  |  | 22/30 |  |  |  |  |
|  | Grooming | 1.0 | 0.0 | - | 3.0 | 1.0 | 1.0 | - | 2.0 |  | 1.0 | 0.0 | - | 2.0 |  |
|  | Occ SAP | 10/20 |  |  |  | 11/21 |  |  |  |  | 13/30 |  |  |  |  |
|  | SAP | 0.5 | 0.0 | - | 1.0 | 1.0 | 0.0 | - | 2.0 |  | 0.0 | 0.0 | - | 1.0 |  |
|  | Occ urine | 12/20 |  |  |  | 16/21 |  |  |  |  | 24/30 |  |  |  |  |
|  | Urine | 1.0 | 0.0 | - | 1.0 | 1.0 | 1.0 | - | 1.0 |  | 1.0 | 1.0 | - | 2.0 |  |
|  | Occ boli | 13/20 |  |  |  | 11/21 |  |  |  |  | 15/30 |  |  |  |  |
|  | Boli | 1.0 | 0.0 | - | 2.5 | 1.0 | 0.0 | - | 3.0 |  | 0.5 | 0.0 | - | 4.0 |  |
|  | Body weight | 84.8 | 76.6 | - | 95.2 | 88.3 | 77.2 | - | 91.7 |  | 81.1 | 69.1 | - | 90.6 |  |
| Occurrence (Occ) is shown for the zones and behaviors that were not visited/performed by all animals. *p<0.05 compared to AFR, +p<0.05 compared to MS15 (Mann-Whitney U test).  Abbreviations: BE, bridge entrance; corr, corridor; CTRCI, central circle; DCR, dark corner room; D, duration (s); D/F, duration per visit (s); F, frequency; L, latency (s); SAP, stretched attend posture; TOTACT, total activity. | | | | | | | | | | | | | | | |
