# Supplementary material for: Behavioral Profiling in Early Adolescence and Early Adulthood of Male Wistar Rats After Short and Prolonged Maternal Separation
Source: Front Behav Neurosci. 2020 Mar 19;14:37. doi: 10.3389/fnbeh.2020.00037 (PMC7096550; doi:10.3389/fnbeh.2020.00037)
Supplement: Supplementary file 3 [file Table_2.DOCX]

Supplementary Table 2. Results from the second MCSF trial in animals reared according to the three rearing conditions: AFR (n=19), MS15 (n=21) and MS360 (n=28).

|  |  | **AFR** | | | | **MS15** | | | | | **MS360** | | | |  |
| --- | --- | --- | --- | --- | --- | --- | --- | --- | --- | --- | --- | --- | --- | --- | --- |
|  |  | Median | Quartiles | | | Median | Quartiles | | | p-value | Median | Quartiles | | | p-value |
| **Trend analysis** | General activity | 143.5 | 118.0 | - | 209.5 | 190.0 | 112.5 | - | 256.0 |  | 172.3 | 82.8 | - | 256.3 |  |
|  | Exploratory activity | 156.5 | 125.5 | - | 188.5 | 189.5 | 143.5 | - | 226.5 |  | 190.0 | 143.8 | - | 220.3 |  |
|  | Risk assessment | 231.0 | 210.5 | - | 283.0 | 277.0 | 170.5 | - | 300.0 |  | 268.5 | 202.0 | - | 291.0 |  |
|  | Risk taking | 189.0 | 138.0 | - | 249.0 | 224.0 | 151.5 | - | 288.5 |  | 232.3 | 149.8 | - | 273.0 |  |
|  | Shelter seeking | 101.5 | 55.0 | - | 152.0 | 106.5 | 53.0 | - | 128.5 |  | 111.5 | 72.5 | - | 142.0 |  |
| **Center** | L leave | 4.5 | 3.9 | - | 6.1 | 4.4 | 3.2 | - | 7.8 |  | 5.8 | 2.8 | - | 8.8 |  |
|  | F center | 11.0 | 9.0 | - | 14.0 | 13.0 | 8.0 | - | 17.0 |  | 10.0 | 7.0 | - | 18.0 |  |
|  | D center | 113.2 | 54.7 | - | 139.5 | 113.5 | 92.9 | - | 139.1 |  | 102.0 | 50.8 | - | 126.5 |  |
|  | D/F center | 7.9 | 6.0 | - | 11.6 | 8.7 | 6.6 | - | 11.6 |  | 7.9 | 6.2 | - | 12.2 |  |
|  | Distance center | 1440.0 | 1049.8 | - | 1785.1 | 1521.5 | 1260.2 | - | 1999.6 |  | 1472.7 | 905.3 | - | 1852.1 |  |
|  | Velocity center | 11.0 | 9.8 | - | 12.4 | 11.5 | 9.1 | - | 13.7 |  | 11.3 | 8.2 | - | 13.0 |  |
|  | %F center | 14.9 | 13.5 | - | 17.5 | 13.8 | 12.7 | - | 16.7 |  | 14.1 | 10.6 | - | 16.5 |  |
|  | %D center | 9.4 | 4.5 | - | 11.6 | 9.4 | 7.7 | - | 11.6 |  | 8.4 | 4.2 | - | 10.5 |  |
| **Central circle** | L CTRCI | 172.6 | 78.1 | - | 783.7 | 311.0 | 62.5 | - | 384.3 |  | 150.8 | 27.7 | - | 508.6 |  |
|  | F CTRCI | 1.0 | 0.0 | - | 2.0 | 2.0 | 0.0 | - | 3.0 |  | 1.0 | 0.0 | - | 2.0 |  |
|  | D CTRCI | 0.9 | 0.0 | - | 3.7 | 3.0 | 0.0 | - | 5.4 |  | 1.7 | 0.0 | - | 3.6 |  |
|  | D/F CTRCI | 1.3 | 0.7 | - | 1.6 | 1.5 | 1.3 | - | 2.6 |  | 1.2 | 0.9 | - | 2.1 |  |
|  | Distance CTRCI | 21.7 | 2.1 | - | 39.0 | 32.7 | 0.0 | - | 59.3 |  | 29.4 | 0.0 | - | 55.6 |  |
|  | Velocity CTRCI | 19.7 | 13.7 | - | 31.8 | 16.9 | 11.4 | - | 21.2 |  | 18.2 | 12.7 | - | 23.9 |  |
|  | %F CTRCI | 1.1 | 0.0 | - | 3.3 | 1.7 | 0.0 | - | 3.2 |  | 1.6 | 0.0 | - | 2.5 |  |
|  | %D CTRCI | 0.1 | 0.0 | - | 0.3 | 0.2 | 0.0 | - | 0.4 |  | 0.1 | 0.0 | - | 0.3 |  |
|  | Occ CTRCI | 12/19 |  |  |  | 14/21 |  |  |  |  | 20/28 |  |  |  |  |
| **Total corridor** | F total corr | 26.0 | 24.0 | - | 35.0 | 33.0 | 25.0 | - | 42.0 |  | 33.0 | 21.5 | - | 41.0 |  |
|  | D total corr | 446.0 | 342.8 | - | 577.8 | 448.4 | 371.5 | - | 519.7 |  | 403.7 | 331.2 | - | 460.4 |  |
|  | D/F total corr | 16.1 | 11.4 | - | 19.2 | 14.7 | 9.6 | - | 18.9 |  | 11.5 | 9.0 | - | 16.3 |  |
|  | %F total corr | 39.2 | 37.6 | - | 42.9 | 36.7 | 35.2 | - | 41.8 |  | 39.2 | 36.3 | - | 43.3 |  |
|  | %D total corr | 37.0 | 28.4 | - | 47.9 | 37.2 | 30.8 | - | 43.2 |  | 33.4 | 27.5 | - | 38.2 |  |
|  | Occ corrA | 19/19 |  |  |  | 18/21 |  | - |  |  | 25/28 |  | - |  |  |
|  | Occ corrB | 18/19 |  |  |  | 19/21 |  | - |  |  | 26/28 |  | - |  |  |
|  | Occ corrC | 18/19 |  |  |  | 20/21 |  | - |  |  | 27/28 |  | - |  |  |
| **Dark corner**  **room** | L DCR | 27.3 | 12.5 | - | 279.8 | 58.7 | 18.0 | - | 325.6 |  | 137.1 | 16.6 | - | 224.6 |  |
|  | F DCR | 6.0 | 3.0 | - | 10.0 | 8.0 | 5.0 | - | 10.0 |  | 7.0 | 3.0 | - | 11.0 |  |
|  | D DCR | 119.7 | 52.9 | - | 357.8 | 142.3 | 51.6 | - | 202.8 |  | 157.7 | 77.0 | - | 327.1 |  |
|  | D/F DCR | 26.1 | 12.7 | - | 54.8 | 19.8 | 15.5 | - | 26.4 |  | 21.9 | 16.1 | - | 27.8 |  |
|  | %F DCR | 7.1 | 4.1 | - | 13.5 | 7.1 | 5.3 | - | 10.0 |  | 6.9 | 4.5 | - | 9.9 |  |
|  | %D DCR | 9.9 | 4.4 | - | 29.7 | 11.8 | 4.3 | - | 16.8 |  | 13.1 | 6.4 | - | 27.2 |  |
|  | Occ DCR | 19/19 |  |  |  | 18/21 |  | - |  |  | 25/28 |  | - |  |  |
| **Hurdle** | L hurdle | 41.9 | 13.8 | - | 141.1 | 50.3 | 12.6 | - | 107.9 |  | 47.4 | 8.7 | - | 136.2 |  |
|  | F hurdle | 7.0 | 5.0 | - | 10.0 | 10.0 | 7.0 | - | 12.0 |  | 8.5 | 7.0 | - | 13.0 |  |
|  | D hurdle | 116.6 | 72.0 | - | 168.7 | 144.8 | 113.1 | - | 188.1 |  | 143.9 | 96.9 | - | 185.1 |  |
|  | D/F hurdle | 12.7 | 10.7 | - | 22.2 | 14.9 | 13.3 | - | 20.5 |  | 15.1 | 10.8 | - | 18.6 |  |
|  | %F hurdle | 9.5 | 8.8 | - | 12.3 | 9.5 | 8.9 | - | 12.8 |  | 11.2 | 8.6 | - | 15.4 |  |
|  | %D hurdle | 9.7 | 6.0 | - | 14.0 | 12.0 | 9.4 | - | 15.6 |  | 11.9 | 8.0 | - | 15.3 |  |
|  | Occ hurdle | 18/19 |  |  |  | 20/21 |  |  |  |  | 27/28 |  |  |  |  |
| **Slope** | L slope | 46.5 | 34.9 | - | 121.4 | 33.1 | 14.8 | - | 151.6 |  | 41.9 | 17.9 | - | 116.2 |  |
|  | F slope | 9.0 | 5.0 | - | 12.0 | 12.0 | 6.0 | - | 16.0 |  | 9.5 | 6.5 | - | 13.5 |  |
|  | D slope | 96.5 | 70.1 | - | 142.9 | 72.5 | 35.4 | - | 102.6 |  | 112.3 | 84.3 | - | 134.4 |  |
|  | D/F slope | 11.0 | 8.1 | - | 15.5 | 6.7 | 5.0 | - | 8.7 | ** | 10.6 | 7.3 | - | 16.5 | ++ |
|  | %F slope | 11.6 | 9.1 | - | 13.4 | 12.8 | 6.3 | - | 14.5 |  | 12.1 | 10.0 | - | 14.7 |  |
|  | %D slope | 8.0 | 5.8 | - | 11.8 | 6.0 | 2.9 | - | 8.5 |  | 9.3 | 7.0 | - | 11.1 |  |
|  | Occ slope | 17/19 |  |  |  | 19/21 |  |  |  |  | 27/28 |  |  |  |  |
| **Bridge entrance** | L BE | 125.8 | 51.6 | - | 237.3 | 37.5 | 21.9 | - | 125.2 |  | 77.6 | 28.6 | - | 239.2 |  |
|  | F BE | 7.0 | 2.0 | - | 11.0 | 10.0 | 5.0 | - | 13.0 |  | 7.0 | 3.5 | - | 10.0 |  |
|  | D BE | 35.0 | 10.9 | - | 50.9 | 49.0 | 31.5 | - | 77.1 |  | 32.7 | 9.0 | - | 48.8 |  |
|  | D/F BE | 5.2 | 4.8 | - | 5.8 | 5.4 | 3.9 | - | 8.1 |  | 5.0 | 3.1 | - | 6.9 |  |
|  | %F BE | 9.0 | 3.5 | - | 11.9 | 9.6 | 5.6 | - | 12.5 |  | 8.0 | 4.8 | - | 10.1 |  |
|  | %D BE | 2.9 | 0.9 | - | 4.2 | 4.1 | 2.6 | - | 6.4 |  | 2.7 | 0.7 | - | 4.0 |  |
|  | Occ BE | 17/19 |  |  |  | 19/21 |  |  |  |  | 27/28 |  |  |  |  |
| **Bridge** | L bridge | 112.3 | 65.7 | - | 164.8 | 44.2 | 26.0 | - | 133.0 |  | 100.2 | 35.1 | - | 265.2 |  |
|  | F bridge | 4.0 | 1.0 | - | 6.0 | 5.0 | 2.0 | - | 6.0 |  | 4.0 | 1.5 | - | 5.0 |  |
|  | D bridge | 112.3 | 19.7 | - | 163.3 | 122.7 | 44.6 | - | 147.6 |  | 115.9 | 81.8 | - | 157.0 |  |
|  | D/F bridge | 31.8 | 19.7 | - | 41.6 | 22.3 | 20.6 | - | 33.6 |  | 33.7 | 23.1 | - | 63.2 |  |
|  | %F bridge | 4.5 | 1.8 | - | 5.9 | 4.3 | 2.8 | - | 6.0 |  | 4.0 | 2.4 | - | 5.1 |  |
|  | %D bridge | 9.3 | 1.6 | - | 13.6 | 10.2 | 3.7 | - | 12.2 |  | 9.6 | 6.8 | - | 13.0 |  |
|  | Occ bridge | 15/19 |  |  |  | 19/21 |  |  |  |  | 26/28 |  |  |  |  |
| **Activity** | TOTACT | 73.0 | 56.0 | - | 91.0 | 90.0 | 68.0 | - | 113.0 |  | 81.5 | 58.5 | - | 110.5 |  |
|  | Distance total | 5957.4 | 4350.1 | - | 6830.5 | 6342.8 | 5356.0 | - | 7224.6 |  | 5628.7 | 4283.5 | - | 6905.3 |  |
|  | Velocity mean | 6.8 | 5.2 | - | 7.4 | 6.9 | 6.5 | - | 8.5 |  | 7.1 | 5.3 | - | 8.3 |  |
|  | Rearing | 43.0 | 32.0 | - | 58.0 | 55.0 | 47.0 | - | 65.0 |  | 43.5 | 32.5 | - | 62.0 |  |
|  | Occ all zones visited | 12/19 |  |  |  | 14/21 |  |  |  |  | 20/28 |  |  |  |  |
| **Miscellaneous** | Occ nose poke | 16/19 |  |  |  | 18/21 |  |  |  |  | 25/28 |  |  |  |  |
|  | Nose poke | 4.0 | 1.0 | - | 12.0 | 8.0 | 2.0 | - | 11.0 |  | 6.0 | 2.0 | - | 13.0 |  |
|  | Occ grooming | 13/19 |  |  |  | 17/21 |  |  |  |  | 20/28 |  |  |  |  |
|  | Grooming | 1.0 | 0.0 | - | 4.0 | 2.0 | 1.0 | - | 3.0 |  | 1.0 | 0.0 | - | 3.0 |  |
|  | Occ SAP | 7/19 |  |  |  | 7/21 |  |  |  |  | 17/28 |  |  |  |  |
|  | SAP | 0.0 | 0.0 | - | 1.0 | 0.0 | 0.0 | - | 1.0 |  | 1.0 | 0.0 | - | 2.0 | + |
|  | Occ urine | 10/19 |  |  |  | 15/21 |  |  |  |  | 24/28 |  |  |  | # |
|  | Urine | 1.0 | 0.0 | - | 1.0 | 2.0 | 0.0 | - | 3.0 |  | 2.0 | 1.0 | - | 2.5 |  |
|  | Occ boli | 1/19 |  |  |  | 1/21 |  |  |  |  | 2/28 |  |  |  |  |
|  | Boli | 0.0 | 0.0 | - | 0.0 | 0.0 | 0.0 | - | 0.0 |  | 0.0 | 0.0 | - | 0.0 |  |
|  | Body weight | 301.4 | 282.9 | - | 316.9 | 306.9 | 294.4 | - | 321.7 |  | 289.3 | 275.3 | - | 303.6 | * |
| Occurrence (Occ) is shown for the zones and behaviors that were not visited/performed by all animals. **p<0.01 compared to AFR, +p<0.05, ++p<0.01 compared to MS15 (Mann-Whitney U test); #p<0.05 compared to AFR (Maximum-Likelihood Chi^2^ test).  Abbreviations: BE, bridge entrance; corr, corridor; CTRCI, central circle; DCR, dark corner room; D, duration (s); D/F, duration per visit (s); F, frequency; L, latency (s); SAP, stretched attend posture; TOTACT, total activity. | | | | | | | | | | | | | | | |
