# Supplementary material for: Behavioral Profiling in Early Adolescence and Early Adulthood of Male Wistar Rats After Short and Prolonged Maternal Separation
Source: Front Behav Neurosci. 2020 Mar 19;14:37. doi: 10.3389/fnbeh.2020.00037 (PMC7096550; doi:10.3389/fnbeh.2020.00037)
Supplement: Supplementary file 4 [file Table_3.DOCX]

Supplementary Table 3. Results from the first MCSF trial in animals classified into the Main type (n=42), Explorer (n=9) or Shelter seeker (n=20) behavioral type.

|  |  | **Main type** | | | | **Explorers** | | | | | **Shelter seekers** | | | | | |
| --- | --- | --- | --- | --- | --- | --- | --- | --- | --- | --- | --- | --- | --- | --- | --- | --- |
|  |  | Median | Quartiles | | | Median | Quartiles | | | p-value | Median | Quartiles | | | p-value |  |
| **Type scores** | Exploration | 115.0 | 90.0 | - | 140.0 | 165.0 | 156.0 | - | 175.0 | *** | 60.5 | 50.5 | - | 82.5 | ***. +++ |  |
|  | Shelter seeking | 317.3 | 276.0 | - | 375.0 | 187.0 | 178.5 | - | 207.0 | *** | 453.5 | 427.8 | - | 497.0 | ***. +++ |  |
| **Testing order** | Beginning | 24/42 |  |  |  |  | 1/9 |  |  | ## | 14/20 |  |  |  | @@ |  |
| **Center** | L leave | 25.1 | 11.7 | - | 49.6 | 26.5 | 14.0 | - | 42.7 |  | 11.3 | 6.9 | - | 39.9 |  |  |
|  | F center | 9.0 | 7.0 | - | 12.0 | 18.0 | 13.0 | - | 20.0 | *** | 3.0 | 2.0 | - | 4.0 | ***. +++ |  |
|  | D center | 103.6 | 73.9 | - | 147.2 | 151.0 | 146.6 | - | 164.5 | ** | 44.7 | 18.6 | - | 75.0 | ***. ++ |  |
|  | D/F center | 11.3 | 9.3 | - | 16.4 | 10.4 | 8.4 | - | 12.2 |  | 14.8 | 9.3 | - | 23.9 |  |  |
|  | Distance center | 1237.5 | 941.4 | - | 1603.0 | 1880.3 | 1802.6 | - | 2058.3 | *** | 487.5 | 187.0 | - | 819.7 | ***. +++ |  |
|  | Velocity center | 9.9 | 8.3 | - | 10.8 | 11.2 | 10.9 | - | 11.6 | * | 7.0 | 5.3 | - | 8.2 | ***. +++ |  |
|  | %F center | 14.0 | 12.2 | - | 17.5 | 16.5 | 14.1 | - | 19.6 |  | 18.2 | 12.3 | - | 24.3 |  |  |
|  | %D center | 8.6 | 6.1 | - | 12.2 | 12.6 | 12.2 | - | 13.7 | ** | 3.7 | 1.5 | - | 6.2 | ***. ++ |  |
| **Central circle** | L CTRCI | 255.0 | 113.9 | - | 518.6 | 127.5 | 83.9 | - | 231.1 |  | 67.0 | 29.0 | - | 166.5 |  |  |
|  | F CTRCI | 1.5 | 1.0 | - | 2.0 | 5.0 | 4.0 | - | 6.0 | *** | 0.0 | 0.0 | - | 1.0 | ***. +++ |  |
|  | D CTRCI | 3.0 | 1.4 | - | 4.0 | 6.3 | 3.0 | - | 8.3 | * | 0.0 | 0.0 | - | 0.8 | ***. +++ |  |
|  | D/F CTRCI | 2.0 | 1.4 | - | 2.6 | 1.5 | 0.6 | - | 1.6 |  | 1.2 | 0.8 | - | 3.4 |  |  |
|  | Distance CTRCI | 28.4 | 3.5 | - | 42.6 | 119.9 | 75.3 | - | 138.3 | *** | 0.0 | 0.0 | - | 0.0 | ***. +++ |  |
|  | Velocity CTRCI | 10.9 | 8.4 | - | 15.7 | 17.6 | 13.5 | - | 32.9 | ** | 7.0 | 3.8 | - | 11.0 | ++ |  |
|  | %F CTRCI | 2.3 | 1.3 | - | 3.2 | 5.5 | 3.9 | - | 5.7 | ** | 0.0 | 0.0 | - | 3.6 | ++ |  |
|  | %D CTRCI | 0.2 | 0.1 | - | 0.3 | 0.5 | 0.2 | - | 0.7 | * | 0.0 | 0.0 | - | 0.1 | ***. +++ |  |
|  | Occ CTRCI | 33/42 |  |  |  | 9/9 |  |  |  | # | 6/20 |  |  |  | ###. @@@ |  |
| **Total corridor** | F total corr | 21.0 | 17.0 | - | 27.0 | 33.0 | 29.0 | - | 36.0 | *** | 6.5 | 4.0 | - | 12.5 | ***. +++ |  |
|  | D total corr | 317.1 | 236.2 | - | 390.3 | 346.0 | 314.7 | - | 396.7 |  | 259.4 | 72.3 | - | 376.8 |  |  |
|  | D/F total corr | 14.2 | 11.3 | - | 19.7 | 9.9 | 9.3 | - | 10.3 | ** | 24.4 | 16.9 | - | 35.7 | ***. +++ |  |
|  | %F total corr | 34.5 | 29.8 | - | 38.4 | 32.5 | 30.8 | - | 33.7 |  | 45.1 | 38.6 | - | 48.3 | ***. ++ |  |
|  | %D total corr | 26.3 | 19.6 | - | 32.4 | 28.7 | 26.1 | - | 33.0 |  | 21.6 | 6.0 | - | 31.4 |  |  |
|  | Occ corrA | 38/42 |  |  |  | 9/9 |  |  |  |  | 11/20 |  |  |  | ##. @@ |  |
|  | Occ corrB | 28/42 |  |  |  | 9/9 |  |  |  |  | 12/20 |  |  |  | ##. @@ |  |
|  | Occ corrC | 42/42 |  |  |  | 9/9 |  |  |  |  | 11/20 |  |  |  | ###. @@ |  |
| **Dark corner room** | L DCR | 187.4 | 79.6 | - | 515.2 | 159.7 | 93.8 | - | 682.5 |  | 109.0 | 16.3 | - | 387.3 |  |  |
|  | F DCR | 5.0 | 3.0 | - | 7.0 | 5.0 | 5.0 | - | 8.0 |  | 1.5 | 0.0 | - | 5.0 | **. ++ |  |
|  | D DCR | 215.8 | 111.0 | - | 371.1 | 120.6 | 104.4 | - | 167.9 |  | 179.3 | 0.0 | - | 791.6 |  |  |
|  | D/F DCR | 45.6 | 28.8 | - | 70.6 | 21.7 | 19.1 | - | 27.9 | *** | 160.2 | 94.4 | - | 239.3 | ***. +++ |  |
|  | %F DCR | 7.2 | 4.8 | - | 10.4 | 5.5 | 4.7 | - | 6.5 |  | 7.5 | 0.0 | - | 28.3 |  |  |
|  | %D DCR | 17.9 | 9.2 | - | 30.8 | 10.0 | 8.7 | - | 14.0 |  | 14.9 | 0.0 | - | 65.9 |  |  |
|  | Occ DCR | 36/42 |  |  |  | 9/9 |  |  |  |  | 11/20 |  |  |  | #. @@ |  |
| **Hurdle** | L hurdle | 252.3 | 75.7 | - | 365.2 | 128.7 | 62.6 | - | 162.9 |  | 104.4 | 45.3 | - | 257.3 |  |  |
|  | F hurdle | 5.0 | 3.0 | - | 6.0 | 8.0 | 6.0 | - | 9.0 | *** | 1.0 | 0.0 | - | 4.0 | ***. +++ |  |
|  | D hurdle | 83.4 | 65.9 | - | 118.9 | 110.2 | 79.9 | - | 132.8 |  | 43.6 | 0.0 | - | 282.2 |  |  |
|  | D/F hurdle | 20.4 | 13.8 | - | 29.1 | 14.4 | 13.3 | - | 14.8 | * | 48.7 | 23.1 | - | 61.3 | **. +++ |  |
|  | %F hurdle | 7.5 | 5.3 | - | 10.0 | 7.3 | 5.7 | - | 10.6 |  | 8.5 | 0.0 | - | 15.7 |  |  |
|  | %D hurdle | 6.9 | 5.5 | - | 9.9 | 9.1 | 6.6 | - | 11.0 |  | 3.6 | 0.0 | - | 23.5 |  |  |
|  | Occ hurdle | 42/42 |  |  |  | 9/9 |  |  |  |  | 12/20 |  |  |  | ###. @@ |  |
| **Slope** | L slope | 211.7 | 70.6 | - | 396.6 | 141.6 | 130.0 | - | 157.6 |  | 62.2 | 34.2 | - | 401.5 |  |  |
|  | F slope | 9.0 | 7.0 | - | 12.0 | 14.0 | 11.0 | - | 16.0 | * | 0.0 | 0.0 | - | 2.5 | ***. +++ |  |
|  | D slope | 144.5 | 92.9 | - | 191.6 | 150.6 | 119.7 | - | 175.2 |  | 0.0 | 0.0 | - | 93.3 | ***. + |  |
|  | D/F slope | 14.3 | 11.5 | - | 20.0 | 10.8 | 9.8 | - | 11.7 | ** | 78.4 | 16.6 | - | 158.7 | **. ++ |  |
|  | %F slope | 15.1 | 11.3 | - | 18.9 | 13.6 | 12.1 | - | 17.0 |  | 0.0 | 0.0 | - | 12.5 | ***. + |  |
|  | %D slope | 12.0 | 7.7 | - | 15.9 | 12.5 | 9.9 | - | 14.5 |  | 0.0 | 0.0 | - | 7.8 | ***. + |  |
|  | Occ slope | 41/42 |  |  |  | 9/9 |  |  |  |  | 4/20 |  |  |  | ###. @@@ |  |
| **Bridge**  **entrance** | L BE | 274.1 | 116.0 | - | 459.8 | 236.1 | 149.4 | - | 285.8 |  | 337.4 | 284.6 | - | 466.4 |  |  |
|  | F BE | 8.0 | 5.0 | - | 11.0 | 12.0 | 11.0 | - | 14.0 | * | 0.0 | 0.0 | - | 0.0 | ***. +++ |  |
|  | D BE | 42.1 | 26.8 | - | 67.4 | 67.2 | 48.1 | - | 89.6 |  | 0.0 | 0.0 | - | 0.0 | ***. +++ |  |
|  | D/F BE | 6.1 | 4.2 | - | 7.9 | 5.3 | 4.0 | - | 6.3 |  | 6.6 | 4.0 | - | 10.0 |  |  |
|  | %F BE | 11.9 | 8.5 | - | 16.9 | 11.9 | 10.3 | - | 14.1 |  | 0.0 | 0.0 | - | 0.0 | ***. +++ |  |
|  | %D BE | 3.5 | 2.2 | - | 5.6 | 5.6 | 4.0 | - | 7.4 |  | 0.0 | 0.0 | - | 0.0 | ***. +++ |  |
|  | Occ BE | 39/42 |  |  |  | 9/9 |  |  |  |  | 3/20 |  |  |  | ###. @@@ |  |
| **Bridge** | L bridge | 323.2 | 162.7 | - | 513.0 | 268.1 | 258.5 | - | 309.7 |  | 455.8 | 344.6 | - | 566.9 |  |  |
|  | F bridge | 4.0 | 2.0 | - | 5.0 | 6.0 | 5.0 | - | 7.0 | ** | 0.0 | 0.0 | - | 0.0 | ***. +++ |  |
|  | D bridge | 135.5 | 66.6 | - | 234.5 | 223.7 | 172.9 | - | 242.9 |  | 0.0 | 0.0 | - | 0.0 | ***. +++ |  |
|  | D/F bridge | 37.8 | 31.9 | - | 54.5 | 33.6 | 30.6 | - | 48.6 |  | 201.2 | 67.7 | - | 334.7 |  |  |
|  | %F bridge | 5.4 | 3.5 | - | 8.1 | 5.6 | 5.3 | - | 7.5 |  | 0.0 | 0.0 | - | 0.0 | ***. +++ |  |
|  | %D bridge | 11.3 | 5.5 | - | 19.5 | 18.6 | 14.4 | - | 20.2 |  | 0.0 | 0.0 | - | 0.0 | ***. +++ |  |
|  | Occ bridge | 37/42 |  |  |  | 9/9 |  |  |  |  | 2/20 |  |  |  | ###. @@@ |  |
| **Activity** | TOTACT | 63.0 | 49.0 | - | 77.0 | 103.0 | 94.0 | - | 107.0 | *** | 18.5 | 9.0 | - | 26.0 | ***. +++ |  |
|  | Distance total | 2195.5 | 1734.7 | - | 2506.2 | 3444.0 | 3201.6 | - | 3865.4 | *** | 1538.9 | 637.5 | - | 1952.7 | ***. +++ |  |
|  | Velocity mean | 7.7 | 6.2 | - | 8.6 | 9.2 | 8.9 | - | 9.4 | ** | 4.7 | 4.1 | - | 6.4 | ***. +++ |  |
|  | Rearing | 37.0 | 24.0 | - | 46.0 | 62.0 | 53.0 | - | 74.0 | *** | 19.5 | 8.5 | - | 25.5 | ***. +++ |  |
|  | Occ all zones visited | 26/42 |  |  |  | 9/9 |  |  |  | ## | 0/20 |  |  |  | ###. @@@ |  |
| **Miscellaneous** | Occ nose poke | 11/42 |  |  |  | 4/9 |  |  |  |  | 3/20 |  |  |  |  |  |
|  | Nose poke | 0.0 | 0.0 | - | 1.0 | 0.0 | 0.0 | - | 2.0 |  | 0.0 | 0.0 | - | 0.0 |  |  |
|  | Occ grooming | 27/42 |  |  |  | 7/9 |  |  |  |  | 16/20 |  |  |  |  |  |
|  | Grooming | 1.0 | 0.0 | - | 2.0 | 1.0 | 1.0 | - | 1.0 |  | 2.0 | 1.0 | - | 4.0 |  |  |
|  | Occ SAP | 21/42 |  |  |  | 3/9 |  |  |  |  | 10/20 |  |  |  |  |  |
|  | SAP | 0.5 | 0.0 | - | 1.0 | 0.0 | 0.0 | - | 1.0 |  | 0.5 | 0.0 | - | 2.0 |  |  |
|  | Occ urine | 31/42 |  |  |  | 8/9 |  |  |  |  | 13/20 |  |  |  |  |  |
|  | Urine | 1.0 | 0.0 | - | 1.0 | 1.0 | 1.0 | - | 1.0 |  | 1.0 | 0.0 | - | 2.5 |  |  |
|  | Occ boli | 20/42 |  |  |  | 4/9 |  |  |  |  | 15/20 |  |  |  |  |  |
|  | Boli | 0.0 | 0.0 | - | 3.0 | 0.0 | 0.0 | - | 1.0 |  | 2.5 | 0.5 | - | 4.0 | *. ++ |  |
|  | Body weight | 82.7 | 72.2 | - | 89.8 | 97.2 | 90.6 | - | 99.7 | *** | 84.3 | 68.2 | - | 89.7 | ++ |  |
| Occurrence (Occ) is shown for the zones and behaviors that were not visited/performed by all animals. *p<0.05, **p<0.01, ***p<0.001 compared to main type, +p<0.05, ++p<0.01, +++p<0.001 compared to explorers (Mann-Whitney U test); #p<0.05, ##p<0.01, ###p<0.001 compared to main type, @@p<0.01, @@@p<0.001 compared to explorers (Maximum-Likelihood Chi^2^ test).  Abbreviations: BE, bridge entrance; corr, corridor; CTRCI, central circle; DCR, dark corner room; D, duration (s); D/F, duration per visit (s); F, frequency; L, latency (s); SAP, stretched attend posture; TOTACT, total activity. | | | | | | | | | | | | | | | | |
