# Supplementary material for: Behavioral Profiling in Early Adolescence and Early Adulthood of Male Wistar Rats After Short and Prolonged Maternal Separation
Source: Front Behav Neurosci. 2020 Mar 19;14:37. doi: 10.3389/fnbeh.2020.00037 (PMC7096550; doi:10.3389/fnbeh.2020.00037)
Supplement: Supplementary file 5 [file Table_4.DOCX]

Supplementary Table 4. Results from the second MCSF trial in animals classified into the Main type (n=39), Explorer (n=8) or Shelter seeker (n=20) behavioral type.

|  |  | **Main type** | | | | **Explorers** | | | | | **Shelter seekers** | | | | |
| --- | --- | --- | --- | --- | --- | --- | --- | --- | --- | --- | --- | --- | --- | --- | --- |
|  |  | Median | Quartiles | | | Median | Quartiles | | | p-value | Median | Quartiles | | | p-value |
| **Trend analysis** | General activity | 197.5 | 122.5 | - | 234.5 | 221.3 | 153.3 | - | 319.3 |  | 114.0 | 43.8 | - | 167.5 | **. + |
|  | Exploratory activity | 189.5 | 137.0 | - | 226.0 | 205.3 | 175.0 | - | 215.3 |  | 150.0 | 111.0 | - | 186.3 | *. + |
|  | Risk assessment | 271.5 | 221.5 | - | 305.5 | 262.0 | 233.0 | - | 290.3 |  | 188.3 | 144.0 | - | 275.3 | ** |
|  | Risk taking | 233.5 | 155.5 | - | 268.0 | 288.8 | 236.3 | - | 302.5 |  | 166.5 | 62.0 | - | 220.3 | *. ++ |
|  | Shelter seeking | 115.0 | 70.5 | - | 152.0 | 96.0 | 75.0 | - | 111.5 |  | 96.5 | 26.5 | - | 150.5 |  |
| **Center** | L leave | 4.1 | 2.8 | - | 5.8 | 3.9 | 2.1 | - | 5.1 |  | 8.1 | 5.7 | - | 16.3 | ***. ++ |
|  | F center | 12.0 | 9.0 | - | 16.0 | 14.0 | 11.0 | - | 23.5 |  | 9.0 | 2.5 | - | 14.0 | *. + |
|  | D center | 106.6 | 59.4 | - | 124.4 | 145.4 | 80.2 | - | 154.0 |  | 109.6 | 39.1 | - | 138.5 |  |
|  | D/F center | 7.9 | 6.2 | - | 11.4 | 7.4 | 6.3 | - | 9.5 |  | 11.6 | 6.4 | - | 15.8 |  |
|  | Distance center | 1519.2 | 1080.0 | - | 1981.3 | 1928.9 | 1183.3 | - | 2674.7 |  | 1339.6 | 510.0 | - | 1633.2 |  |
|  | Velocity center | 11.6 | 10.6 | - | 13.5 | 12.1 | 10.8 | - | 14.0 |  | 9.3 | 7.2 | - | 11.5 | **. + |
|  | %F center | 13.8 | 10.9 | - | 16.5 | 14.9 | 13.6 | - | 17.3 |  | 15.2 | 9.1 | - | 16.9 |  |
|  | %D center | 8.8 | 4.9 | - | 10.3 | 12.0 | 6.7 | - | 12.8 |  | 9.1 | 3.3 | - | 11.5 |  |
| **Central circle** | L CTRCI | 146.7 | 33.8 | - | 431.3 | 196.7 | 89.5 | - | 568.4 |  | 195.2 | 62.5 | - | 357.4 |  |
|  | F CTRCI | 1.0 | 0.0 | - | 2.0 | 1.5 | 1.0 | - | 3.5 |  | 0.5 | 0.0 | - | 2.0 |  |
|  | D CTRCI | 2.3 | 0.0 | - | 4.4 | 3.4 | 2.5 | - | 4.7 |  | 0.3 | 0.0 | - | 1.9 | + |
|  | D/F CTRCI | 1.4 | 1.2 | - | 2.1 | 1.6 | 1.1 | - | 3.7 |  | 1.0 | 0.6 | - | 1.6 |  |
|  | Distance CTRCI | 31.6 | 10.5 | - | 55.8 | 32.7 | 22.0 | - | 128.4 |  | 3.1 | 0.0 | - | 47.6 |  |
|  | Velocity CTRCI | 19.1 | 12.8 | - | 21.9 | 15.7 | 10.2 | - | 22.6 |  | 18.8 | 11.4 | - | 23.9 |  |
|  | %F CTRCI | 1.5 | 0.0 | - | 3.0 | 1.9 | 0.9 | - | 3.0 |  | 0.7 | 0.0 | - | 3.0 |  |
|  | %D CTRCI | 0.2 | 0.0 | - | 0.4 | 0.3 | 0.2 | - | 0.4 |  | 0.0 | 0.0 | - | 0.2 | + |
|  | Occ CTRCI | 29/39 |  |  |  | 7/8 |  |  |  |  | 10/20 |  |  |  |  |
| **Total corridor** | F total corr | 34.0 | 26.0 | - | 41.0 | 36.0 | 29.5 | - | 50.5 |  | 24.0 | 13.5 | - | 31.5 | **. + |
|  | D total corr | 437.5 | 364.8 | - | 488.8 | 415.2 | 394.6 | - | 461.9 |  | 427.7 | 271.7 | - | 587.3 |  |
|  | D/F total corr | 12.2 | 10.3 | - | 16.5 | 11.9 | 8.9 | - | 14.7 |  | 18.6 | 10.2 | - | 23.9 | * |
|  | %F total corr | 38.5 | 34.7 | - | 40.3 | 36.7 | 35.6 | - | 39.1 |  | 42.4 | 37.5 | - | 44.4 | *. + |
|  | %D total corr | 36.3 | 30.3 | - | 40.5 | 34.4 | 32.7 | - | 38.3 |  | 35.5 | 22.6 | - | 48.8 |  |
|  | Occ corrA | 38/39 |  |  |  | 8/8 |  |  |  |  | 15/20 |  |  |  | ## |
|  | Occ corrB | 37/39 |  |  |  | 8/8 |  |  |  |  | 17/20 |  |  |  |  |
|  | Occ corrC | 38/39 |  |  |  | 8/8 |  |  |  |  | 18/20 |  |  |  |  |
| **Dark corner**  **room** | L DCR | 56.9 | 14.8 | - | 285.6 | 60.7 | 28.1 | - | 195.6 |  | 31.8 | 13.1 | - | 242.2 |  |
|  | F DCR | 7.0 | 3.0 | - | 9.0 | 8.0 | 6.5 | - | 10.5 |  | 5.0 | 0.5 | - | 10.5 |  |
|  | D DCR | 170.8 | 61.7 | - | 290.1 | 119.2 | 86.0 | - | 156.3 |  | 121.1 | 9.8 | - | 346.0 |  |
|  | D/F DCR | 22.7 | 16.1 | - | 32.6 | 11.8 | 11.1 | - | 16.6 |  | 20.0 | 14.6 | - | 56.1 |  |
|  | %F DCR | 6.9 | 4.5 | - | 10.0 | 7.7 | 6.2 | - | 8.9 |  | 6.9 | 1.4 | - | 15.8 |  |
|  | %D DCR | 14.2 | 5.1 | - | 24.0 | 9.9 | 7.1 | - | 12.9 |  | 10.0 | 0.8 | - | 28.7 |  |
|  | Occ DCR | 38/39 |  |  |  | 8/8 |  |  |  |  | 15/20 |  |  |  | ## |
| **Hurdle** | L hurdle | 46.3 | 9.6 | - | 121.4 | 47.3 | 23.6 | - | 60.5 |  | 56.8 | 16.3 | - | 141.1 |  |
|  | F hurdle | 10.0 | 7.0 | - | 13.0 | 10.0 | 8.5 | - | 13.0 |  | 7.0 | 5.5 | - | 8.5 | ++ |
|  | D hurdle | 142.5 | 106.6 | - | 188.1 | 178.2 | 156.8 | - | 186.7 |  | 108.8 | 67.5 | - | 157.9 | + |
|  | D/F hurdle | 15.0 | 10.9 | - | 18.8 | 15.2 | 14.0 | - | 18.5 |  | 14.0 | 10.8 | - | 20.7 |  |
|  | %F hurdle | 10.8 | 8.8 | - | 12.8 | 9.7 | 9.1 | - | 11.3 |  | 10.5 | 8.7 | - | 17.2 |  |
|  | %D hurdle | 11.8 | 8.9 | - | 15.6 | 14.8 | 13.0 | - | 15.4 |  | 9.0 | 5.6 | - | 13.1 | + |
|  | Occ hurdle | 38/39 |  |  |  | 8/8 |  |  |  |  | 18/20 |  |  |  |  |
| **Slope** | L slope | 35.2 | 15.4 | - | 92.3 | 30.5 | 14.8 | - | 57.2 |  | 52.9 | 34.9 | - | 188.3 |  |
|  | F slope | 12.0 | 9.0 | - | 13.0 | 13.0 | 7.5 | - | 15.0 |  | 5.0 | 3.5 | - | 10.0 | ***. + |
|  | D slope | 105.1 | 81.2 | - | 137.6 | 88.2 | 71.1 | - | 113.5 |  | 69.5 | 29.4 | - | 122.5 | * |
|  | D/F slope | 9.9 | 7.3 | - | 12.9 | 7.6 | 5.3 | - | 9.5 |  | 8.5 | 6.7 | - | 15.7 |  |
|  | %F slope | 12.7 | 11.1 | - | 14.9 | 11.5 | 9.7 | - | 12.9 |  | 10.4 | 5.7 | - | 14.2 |  |
|  | %D slope | 8.7 | 6.7 | - | 11.4 | 7.3 | 5.9 | - | 9.4 |  | 5.8 | 2.4 | - | 10.2 | * |
|  | Occ slope | 38/39 |  |  |  | 8/8 |  |  |  |  | 17/20 |  |  |  |  |
| **Bridge**  **entrance** | L BE | 74.7 | 37.0 | - | 145.2 | 25.8 | 16.3 | - | 147.3 |  | 69.5 | 26.3 | - | 439.6 |  |
|  | F BE | 8.0 | 6.0 | - | 11.0 | 11.0 | 7.5 | - | 13.5 |  | 2.5 | 1.0 | - | 9.0 | **. ++ |
|  | D BE | 42.8 | 31.5 | - | 54.4 | 47.7 | 39.7 | - | 70.0 |  | 10.9 | 8.0 | - | 31.6 | **. ++ |
|  | D/F BE | 5.0 | 3.9 | - | 5.8 | 5.8 | 4.1 | - | 7.2 |  | 5.8 | 3.7 | - | 7.1 |  |
|  | %F BE | 9.4 | 7.8 | - | 12.7 | 9.4 | 9.1 | - | 10.9 |  | 5.0 | 3.5 | - | 9.1 | **. + |
|  | %D BE | 3.6 | 2.6 | - | 4.5 | 4.0 | 3.3 | - | 5.8 |  | 0.9 | 0.7 | - | 2.6 | **. ++ |
|  | Occ BE | 38/39 |  |  |  | 8/8 |  |  |  |  | 17/20 |  |  |  |  |
| **Bridge** | L bridge | 77.7 | 44.2 | - | 144.2 | 29.4 | 18.3 | - | 152.8 |  | 139.6 | 39.6 | - | 576.8 |  |
|  | F bridge | 4.0 | 3.0 | - | 5.0 | 5.5 | 4.0 | - | 6.0 |  | 1.0 | 0.5 | - | 4.5 | **. + |
|  | D bridge | 116.4 | 82.0 | - | 149.5 | 164.3 | 113.7 | - | 224.2 |  | 39.4 | 1.8 | - | 147.3 | *. + |
|  | D/F bridge | 31.0 | 21.4 | - | 38.8 | 37.2 | 21.7 | - | 51.5 |  | 24.5 | 22.3 | - | 52.9 |  |
|  | %F bridge | 4.5 | 3.4 | - | 6.0 | 4.6 | 4.4 | - | 5.6 |  | 2.7 | 0.7 | - | 4.6 | **. + |
|  | %D bridge | 9.6 | 6.8 | - | 12.4 | 13.6 | 9.4 | - | 18.6 |  | 3.3 | 0.1 | - | 12.2 | *. + |
|  | Occ bridge | 37/39 |  |  |  | 8/8 |  |  |  |  | 15/20 |  |  |  | # |
| **Activity** | TOTACT | 90.0 | 69.0 | - | 103.0 | 96.0 | 79.0 | - | 137.0 |  | 59.0 | 31.0 | - | 76.0 | **. + |
|  | Distance total | 6005.8 | 5030.7 | - | 6947.6 | 6428.1 | 5042.6 | - | 8327.9 |  | 5307.5 | 3004.6 | - | 6690.7 |  |
|  | Velocity mean | 7.1 | 6.6 | - | 8.2 | 7.3 | 6.9 | - | 8.7 |  | 6.3 | 4.4 | - | 7.0 | *. + |
|  | Rearing | 55.0 | 38.0 | - | 63.0 | 62.5 | 45.0 | - | 74.5 |  | 34.5 | 19.0 | - | 56.5 | **. + |
|  | Occ all zones visited | 29/39 |  |  |  | 7/8 |  |  |  |  | 10/20 |  |  |  |  |
| **Miscellaneous** | Occ nose poke |  |  |  |  | 8/8 |  |  |  |  | 17/20 |  |  |  |  |
|  | Nose poke |  |  | - | 15.0 | 11.0 | 4.5 | - | 16.0 |  | 4.0 | 1.5 | - | 7.5 |  |
|  | Occ grooming | 30/39 |  |  |  | 8/8 |  |  |  | # | 12/20 |  |  |  | @ |
|  | Grooming | 1.0 | 1.0 | - | 4.0 | 1.0 | 1.0 | - | 2.0 |  | 1.0 | 0.0 | - | 3.0 |  |
|  | Occ SAP | 19/39 |  |  |  | 2/8 |  |  |  |  | 9/20 |  |  |  |  |
|  | SAP | 0.0 | 0.0 | - | 1.0 | 0.0 | 0.0 | - | 0.5 |  | 0.0 | 0.0 | - | 1.0 |  |
|  | Occ urine | 26/39 |  |  |  | 6/8 |  |  |  |  | 17/20 |  |  |  |  |
|  | Urine | 1.0 | 0.0 | - | 2.0 | 1.5 | 0.5 | - | 2.5 |  | 2.0 | 1.0 | - | 3.0 |  |
|  | Occ boli | 0/39 |  |  |  | 2/8 |  |  |  | ## | 2/20 |  |  |  | # |
|  | Boli | 0.0 | 0.0 | - | 0.0 | 0.0 | 0.0 | - | 0.5 | ** | 0.0 | 0.0 | - | 0.0 | * |
|  | Body weight | 295.4 | 279.3 | - | 306.9 | 314.0 | 305.6 | - | 319.2 | * | 298.4 | 283.8 | - | 324.6 |  |
| Occurrence (Occ) is shown for the zones and behaviors that were not visited/performed by all animals. *p<0.05, **p<0.01, ***p<0.001 compared to main type, +p<0.05, ++p<0.01 compared to explorers (Mann-Whitney U test); #p<0.05, ##p<0.01 compared to main type, @p<0.05 compared to explorers (Maximum-Likelihood Chi^2^ test).  Abbreviations: BE, bridge entrance; corr, corridor; CTRCI, central circle; DCR, dark corner room; D, duration (s); D/F, duration per visit (s); F, frequency; L, latency (s); SAP, stretched attend posture; TOTACT, total activity. | | | | | | | | | | | | | | | |
