# Supplementary material for: Behavioral Profiling in Early Adolescence and Early Adulthood of Male Wistar Rats After Short and Prolonged Maternal Separation
Source: Front Behav Neurosci. 2020 Mar 19;14:37. doi: 10.3389/fnbeh.2020.00037 (PMC7096550; doi:10.3389/fnbeh.2020.00037)
Supplement: Supplementary file 6 [file Table_5.docx]

Supplementary Table 5. Results from the MCSF in the first (n=71) compared to the second (n=68) trial in the whole cohort.

|  |  | **Trial 1** | | | | **Trial 2** | | | |  |
| --- | --- | --- | --- | --- | --- | --- | --- | --- | --- | --- |
|  |  | Median | Quartiles | | | Median | Quartiles | | | p-value |
| **Center** | L leave | 23.2 | 10.9 | - | 42.7 | 4.7 | 3.2 | - | 8.1 | *** |
|  | F center | 8.0 | 3.0 | - | 12.0 | 11.0 | 8.0 | - | 16.0 | *** |
|  | D center | 100.2 | 52.4 | - | 149.2 | 108.6 | 55.5 | - | 138.5 |  |
|  | D/F center | 12.0 | 8.9 | - | 17.7 | 8.1 | 6.3 | - | 11.8 | *** |
|  | Distance center | 1129.1 | 611.5 | - | 1633.3 | 1456.0 | 989.5 | - | 1941.8 | *** |
|  | Velocity center | 9.3 | 7.4 | - | 10.8 | 11.2 | 9.5 | - | 13.3 | *** |
|  | %F center | 15.4 | 12.3 | - | 19.0 | 14.4 | 11.2 | - | 16.7 |  |
|  | %D center | 8.3 | 4.4 | - | 12.4 | 9.0 | 4.6 | - | 11.5 |  |
| **Central circle** | L CTRCI | 218.7 | 76.9 | - | 450.3 | 175.8 | 62.5 | - | 431.3 |  |
|  | F CTRCI | 1.0 | 0.0 | - | 2.0 | 1.0 | 0.0 | - | 2.0 |  |
|  | D CTRCI | 2.1 | 0.0 | - | 4.0 | 1.8 | 0.0 | - | 4.1 |  |
|  | D/F CTRCI | 1.6 | 1.2 | - | 2.6 | 1.4 | 1.0 | - | 2.0 |  |
|  | Distance CTRCI | 23.0 | 0.0 | - | 45.4 | 26.1 | 0.0 | - | 55.8 |  |
|  | Velocity CTRCI | 11.9 | 9.6 | - | 17.1 | 18.4 | 12.7 | - | 22.6 | * |
|  | %F CTRCI | 2.2 | 0.0 | - | 4.0 | 1.5 | 0.0 | - | 2.8 |  |
|  | %D CTRCI | 0.2 | 0.0 | - | 0.3 | 0.1 | 0.0 | - | 0.3 |  |
|  | Occ CTRCI | 48/71 |  |  |  | 46/68 |  |  |  |  |
| **Total corridor** | F total corr | 19.0 | 12.0 | - | 27.0 | 30.5 | 24.0 | - | 40.0 | *** |
|  | D total corr | 314.4 | 226.9 | - | 390.3 | 436.5 | 355.1 | - | 500.0 | *** |
|  | D/F total corr | 15.1 | 11.2 | - | 21.6 | 13.5 | 10.1 | - | 18.7 | * |
|  | %F total corr | 36.2 | 32.1 | - | 42.4 | 38.7 | 35.6 | - | 42.7 | * |
|  | %D total corr | 26.1 | 18.9 | - | 32.4 | 36.2 | 29.4 | - | 41.5 | *** |
|  | Occ corrA | 58/71 |  |  |  | 62/68 |  |  |  |  |
|  | Occ corrB | 59/71 |  |  |  | 63/68 |  |  |  |  |
|  | Occ corrC | 62/71 |  |  |  | 65/68 |  |  |  |  |
| **Dark corner room** | L DCR | 171.3 | 57.7 | - | 469.8 | 46.9 | 14.1 | - | 242.6 | * |
|  | F DCR | 4.0 | 2.0 | - | 7.0 | 7.0 | 3.0 | - | 10.0 | *** |
|  | D DCR | 169.2 | 56.4 | - | 391.1 | 148.0 | 57.5 | - | 314.4 | * |
|  | D/F DCR | 45.6 | 28.1 | - | 88.8 | 21.3 | 14.6 | - | 32.6 | *** |
|  | %F DCR | 6.5 | 3.2 | - | 10.8 | 7.1 | 4.4 | - | 10.3 |  |
|  | %D DCR | 14.0 | 4.7 | - | 32.6 | 12.3 | 4.8 | - | 26.1 | * |
|  | Occ DCR | 56/71 |  |  |  | 62/68 |  |  |  |  |
| **Hurdle** | L hurdle | 171.3 | 62.6 | - | 331.0 | 48.3 | 13.4 | - | 133.8 | *** |
|  | F hurdle | 5.0 | 2.0 | - | 6.0 | 8.0 | 7.0 | - | 12.0 | *** |
|  | D hurdle | 80.4 | 61.6 | - | 132.8 | 141.6 | 90.8 | - | 180.2 | ** |
|  | D/F hurdle | 20.1 | 14.1 | - | 31.6 | 15.0 | 11.1 | - | 20.1 | *** |
|  | %F hurdle | 7.6 | 5.3 | - | 10.8 | 10.4 | 8.8 | - | 12.7 | ** |
|  | %D hurdle | 6.7 | 5.1 | - | 11.0 | 11.7 | 7.5 | - | 14.9 | ** |
|  | Occ hurdle | 63/71 |  |  |  | 65/68 |  |  |  |  |
| **Slope** | L slope | 157.6 | 62.2 | - | 369.5 | 41.9 | 17.4 | - | 118.1 | *** |
|  | F slope | 8.0 | 3.0 | - | 12.0 | 10.5 | 6.0 | - | 13.0 | ** |
|  | D slope | 129.0 | 50.6 | - | 180.0 | 96.8 | 68.6 | - | 130.5 |  |
|  | D/F slope | 13.5 | 11.4 | - | 20.0 | 9.2 | 6.4 | - | 14.7 | *** |
|  | %F slope | 13.6 | 7.5 | - | 18.4 | 12.0 | 9.2 | - | 14.5 |  |
|  | %D slope | 10.7 | 4.2 | - | 15.0 | 8.0 | 5.7 | - | 10.8 |  |
|  | Occ slope | 57/71 |  |  |  | 63/68 |  |  |  | # |
| **Bridge entrance** | L BE | 274.1 | 126.8 | - | 444.7 | 66.4 | 26.3 | - | 224.1 | *** |
|  | F BE | 6.0 | 0.0 | - | 11.0 | 8.0 | 3.5 | - | 11.0 | * |
|  | D BE | 34.0 | 0.0 | - | 61.9 | 38.0 | 11.6 | - | 53.9 |  |
|  | D/F BE | 6.1 | 4.2 | - | 7.9 | 5.2 | 3.8 | - | 6.5 | * |
|  | %F BE | 10.0 | 0.0 | - | 14.3 | 8.9 | 5.0 | - | 11.4 |  |
|  | %D BE | 2.8 | 0.0 | - | 5.1 | 3.2 | 1.0 | - | 4.5 |  |
|  | Occ BE | 51/71 |  |  |  | 63/68 |  |  |  | ### |
| **Bridge** | L bridge | 303.7 | 163.4 | - | 510.0 | 81.0 | 34.1 | - | 162.4 | *** |
|  | F bridge | 3.0 | 0.0 | - | 5.0 | 4.0 | 1.5 | - | 5.0 | * |
|  | D bridge | 107.3 | 0.0 | - | 221.9 | 115.4 | 50.6 | - | 156.2 |  |
|  | D/F bridge | 37.1 | 31.2 | - | 55.2 | 29.4 | 21.5 | - | 42.3 | * |
|  | %F bridge | 4.0 | 0.0 | - | 6.4 | 4.3 | 2.4 | - | 5.7 |  |
|  | %D bridge | 8.9 | 0.0 | - | 18.5 | 9.6 | 4.2 | - | 12.9 |  |
|  | Occ bridge | 48/71 |  |  |  | 60/68 |  |  |  | ### |
| **Activity** | TOTACT | 57.0 | 32.0 | - | 79.0 | 81.0 | 59.0 | - | 103.5 | *** |
|  | Distance total | 2047.4 | 1564.4 | - | 2955.3 | 5924.4 | 4748.9 | - | 6921.9 | *** |
|  | Velocity mean | 7.2 | 5.5 | - | 8.6 | 6.9 | 6.0 | - | 8.0 |  |
|  | Rearing | 32.0 | 20.0 | - | 49.0 | 53.0 | 35.5 | - | 62.0 | *** |
|  | Occ all zones visited | 35/71 |  |  |  | 46/68 |  |  |  | # |
| **Miscellaneous** | Occ nose poke | 18/71 |  |  |  | 59/68 |  |  |  | ### |
|  | Nose poke | 0.0 | 0.0 | - | 1.0 | 6.0 | 2.0 | - | 12.0 | *** |
|  | Occ grooming | 50/71 |  |  |  | 50/68 |  |  |  |  |
|  | Grooming | 1.0 | 0.0 | - | 2.0 | 1.0 | 0.0 | - | 3.0 |  |
|  | Occ SAP | 34/71 |  |  |  | 31/68 |  |  |  |  |
|  | SAP | 0.0 | 0.0 | - | 1.0 | 0.0 | 0.0 | - | 1.0 |  |
|  | Occ urine | 52/71 |  |  |  | 49/68 |  |  |  |  |
|  | Urine | 1.0 | 0.0 | - | 1.0 | 1.0 | 0.0 | - | 3.0 | ** |
|  | Occ boli | 39/71 |  |  |  | 4/68 |  |  |  | ### |
|  | Boli | 1.0 | 0.0 | - | 3.0 | 0.0 | 0.0 | - | 0.0 | *** |
|  | Body weight | 84.9 | 71.9 | - | 91.7 | 298.6 | 282.8 | - | 314.7 | *** |
| Occurrence is shown for the zones and behaviors that were not visited/performed by all animals. *p<0.05, **p<0.01, ***p<0.001 (Wilcoxon matched pairs test); #p<0.05, ###p<0.001 (McNeamar Chi^2^ test) comparing trail 1 and 2.  Abbreviations: BE, bridge entrance; corr, corridor; CTRCI, central circle; DCR, dark corner room; D, duration (s); D/F, duration per visit (s); F, frequency; L, latency (s); Occ, occurrence; SAP, stretched attend posture; TOTACT, total activity. | | | | | | | | | | |
